# NAC regulates metabolism and cell fate in intestinal stem cells

**DOI:** 10.1126/sciadv.adn9750

**Published:** 2025-01-08

**Authors:** Sofia Ramalho, Ferhat Alkan, Stefan Prekovic, Katarzyna Jastrzebski, Eric Pintó Barberà, Liesbeth Hoekman, Maarten Altelaar, Cecilia de Heus, Nalan Liv, Maria J. Rodríguez-Colman, Mehmet Yilmaz, Rob van der Kammen, Juliette Fedry, Mark C. de Gooijer, Saskia Jacoba Elisabeth Suijkerbuijk, William J. Faller, Joana Silva

**Affiliations:** ^1^ Division of Oncogenomics, Netherlands Cancer Institute, Amsterdam, Netherlands.; ^2^ Center for Molecular Medicine, University Medical Center Utrecht, Utrecht, Netherlands.; ^3^ Division of Molecular Carcinogenesis, Netherlands Cancer Institute, Amsterdam, Netherlands.; ^4^ Proteomics Facility, Netherlands Cancer Institute, Amsterdam, Netherlands.; ^5^ Biomolecular Mass Spectrometry and Proteomics, Bijvoet Center for Biomolecular Research and Utrecht Institute for Pharmaceutical Sciences, Utrecht University, Utrecht, Netherlands.; ^6^ Oncode Institute, Utrecht, Netherlands.; ^7^ MRC Laboratory of Molecular Biology, Cambridge, UK.; ^8^ Division of Pharmacology, Netherlands Cancer Institute, Amsterdam, Netherlands.; ^9^ Faculty of Biology, Medicine and Health, University of Manchester, Manchester, UK.; ^10^ The Christie NHS Foundation Trust, Manchester, UK.; ^11^ Division of Developmental Biology, Institute of Biodynamics and Biocomplexity, Department of Biology, Faculty of Science, Utrecht University, Utrecht, Netherlands.

## Abstract

Intestinal stem cells (ISCs) face the challenge of integrating metabolic demands with unique regenerative functions. Studies have shown an intricate interplay between metabolism and stem cell capacity; however, it is still not understood how this process is regulated. Combining ribosome profiling and CRISPR screening in intestinal organoids, we identify the nascent polypeptide–associated complex (NAC) as a key mediator of this process. Our findings suggest that NAC is responsible for relocalizing ribosomes to the mitochondria and regulating ISC metabolism. Upon NAC inhibition, intestinal cells show decreased import of mitochondrial proteins, which are needed for oxidative phosphorylation, and, consequently, enable the cell to maintain a stem cell identity. Furthermore, we show that overexpression of NACα is sufficient to drive mitochondrial respiration and promote ISC identity. Ultimately, our results reveal the pivotal role of NAC in regulating ribosome localization, mitochondrial metabolism, and ISC function, providing insights into the potential mechanism behind it.

## Teaser

NAC regulates the ribosome localization, metabolic profile, and fate of intestinal stem cells.

## INTRODUCTION

The intestine is a highly dynamic tissue, with a high cell turnover rate. Most intestinal cells live for just 4 to 7 days (*1*) before being shed into the intestinal lumen, and intestinal stem cells (ISCs) are central to the maintenance of this epithelium. The fate of ISCs has been shown to be dependent on their metabolic profile, which is typically characterized by high mitochondrial respiration (*2*). Metabolic dysfunction in ISCs results in a loss of stem cells and can lead to the development of disease (*3*). Furthermore, ISCs are a cell of origin of intestinal cancer (*4*), and considering the crucial role that mitochondrial function plays in cancer maintenance (*5*, *6*), metastasis (*7*, *8*), and acquisition of chemotherapy resistance (*9*–*11*), a better understanding of the processes that regulate metabolism in ISCs could open the door to future therapeutic interventions. Although several studies have addressed the function of mitochondrial metabolism in these cells (*2*, *12*–*14*), the molecular mechanism behind this regulation remains largely unknown.

The regulation of mRNA translation plays a critical role in cellular development and function. Although ribosomes have been thought of as passive machines whose function solely consists of passive protein production, recent work from our group and others has shown that this is not necessarily the case (*15*). Studies have demonstrated that ribosomes can exert a direct regulatory function and that they can play a role as molecular sensors of stress (*16*, *17*). For example, we have shown that in the intestine, ribosomes are crucial in determining the identity of ISCs in the context of amino acid deprivation (*15*).

In this study, we explore how translation regulates metabolism and consequently stem cell identity by combining ribosome profiling and CRISPR screening in mouse intestinal organoids. We use two distinct models of metabolic regulation (ISC differentiation and ribosome impairment) to unveil a central role for the nascent polypeptide–associated complex (NAC) in intestinal cell metabolism. NAC is a highly conserved ribosome-associated complex, whose primary function is to interact with newly synthesized polypeptide chains as they emerge from the ribosome during translation and to assist in their correct folding and targeting (*18*). It is well established that NAC mediates ribosome localization to the endoplasmic reticulum (ER) (*19*), and there have been reports that it can also target ribosomes to the outer membrane of the mitochondria (OMM); however, it is currently unknown whether this OMM targeting is functionally relevant (*20*). Here, we show that NAC plays a crucial role in defining ISC fate, potentially by facilitating the import of peptides into the mitochondria, and thus supporting respiration. Our work goes a step further and establishes NAC as the bridge between translation and metabolism, being translationally regulated itself during ISC differentiation, while also being regulated by ribosome impairment in stress conditions. Our data suggest that the role of NAC in metabolism is mediated by the localization of the ribosome to the OMM and a consequent effect on peptide import. In short, we show that the NAC mediates the localization of ribosomes and plays a central role in ISC maintenance and differentiation.

## RESULTS

### OXPHOS is regulated by mRNA translation in ISCs

**Fig. 1.**
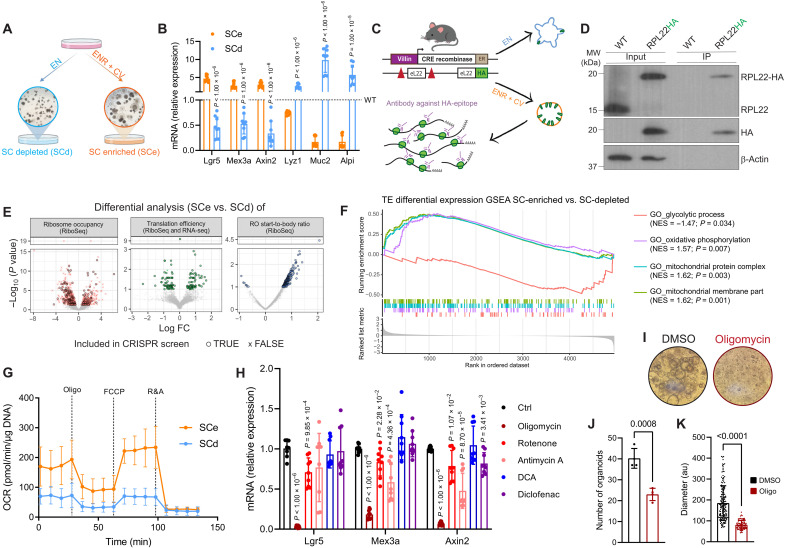
Translation regulation of metabolism affects ISC identity. (**A**) Scheme illustrating the two in vitro models used in this study, highlighting the morphological differences between stem cell–depleted (SCd) and stem cell–enriched (SCe) mouse intestinal organoid cultures. (**B**) Reverse transcription quantitative polymerase chain reaction (RT-qPCR) analysis of genes related to stemness (*Lgr5*, *Mex3a*, and *Axin2*) and differentiation (*Lyz1*, *Muc2*, and *Alpi*) in SCe and SCd organoids compared to wild-type (WT) cultures (dashed line). *Hprt* works as housekeeping control. Means and SD are shown (*n* = 3 biological replicates each in technical triplicates). (**C**) Experimental workflow of the RiboSeq experiment developed to compare the translatome of the different cell populations. (**D**) Western blot analysis confirming efficient pulldown of eL22-HA–tagged ribosomes. β-Actin was used as loading control (*n* = 1 biological replicate). (**E**) Volcano plots for ribosome occupancy (RO), translation efficiency (TE) and RO signal-to-body ratio (S2b) comparing SCe to SCd cultures (*n* = 3 biological replicates). Genes with significant differences between conditions are highlighted, and genes that are selected for follow-up CRISPR dropout screening are marked with “+.” (**F**) Geneset enrichment analysis (GSEA) results based on TE differential expression data comparing SCe to SCd organoids (*n* = 3 biological replicates). (**G**) Oxygen consumption rate (OCR) analysis shows increased respiration in SCe compared to SCd cultures. Means and SD are shown (*n* = 5 biological replicates). (**H**) RT-qPCR analysis of stem-related genes in WT organoids treated with OXPHOS (oligomycin, rotenone, and antimycin A) and glycolysis (dichloroacetate (DCA) and diclofenac) inhibitors for 24 hours. *Hprt* works as housekeeping control. Means and SD are shown (*n* = 3 biological replicates each in technical triplicates). Quantification in fig. S3B. (**I**) Representative images show that oligomycin treatment impairs clonogenic potential of WT ISCs (*n* = 4 biological replicates), which can be measured both by (**J**) final number and (**K**) diameter of organoids. au, arbitrary unit. DMSO, dimethyl sulfoxide.

Previous studies have clearly established the importance of metabolic regulation in ISCs. However, some of this work shows that this regulation appears not to be solely at the transcriptional level (*21*). To develop a deeper understanding of the regulators of metabolism in different intestinal cell types, we generated mouse intestinal organoids that were either enriched for stem cells (SCe) or forced to differentiate (SCd) (*22*) (Fig. 1A). As expected, the SCe cultures acquired a spheroid morphology and showed an increase in various stem cell markers, such as *Lgr5*, *Mex3a*, and *Axin2*, while differentiated cells showed an increase in differentiation markers, such as *Lyz1*, *Muc2*, and *Alpi* (Fig. 1B). We have previously shown that ribosomes play a crucial role in determining the metabolic identity of ISCs in the context of amino acid deprivation (*15*); however, the mechanism behind this process, and its importance outside of stress conditions, remains unknown. We therefore assessed the translatome of SCe and SCd organoids using ribosome profiling (RiboSeq) to gauge the translational regulation of metabolism in normal intestinal physiology.

To do this, we generated SCe and SCd intestinal organoids from mice that express a hemagglutinin (HA)–tagged version of the core ribosomal protein eL22, also known as the RiboTag mouse (Fig. 1C) (*23*). The eL22-HA tag enabled us to efficiently immunoprecipitate ribosomes, from which we could then isolate ribosome-protected mRNA fragments (RPFs) for sequencing (Fig. 1D and fig. S1). This experiment provided information on ribosomal occupancy both in terms of abundance [ribosome occupancy (RO)] and distribution [RO start-to-body ratio (RO s2b)] for each transcript (detailed in Methods). Furthermore, by normalizing the RPFs for each transcript with their overall transcript levels, measured by RNA sequencing (RNA-seq) (fig. S2), we were able to identify genes with differential translation efficiency (TE) between SCe and SCd organoids (Fig. 1E and table S1).

Despite the overall levels of protein synthesis being similar between SCe and SCd cultures (fig. S3A), RiboSeq analysis revealed that around 300 genes are translationally regulated when comparing the two conditions (Fig. 1E and table S1). Most of these hits appear to be regulated by the overall number of ribosomes bound to them (RO). However, we also identified some transcripts that displayed potential changes in initiation and/or elongation rate, as measured by the relative abundance of RPFs at the start of the mRNA compared to the body (RO s2b). Gene set enrichment analysis (GSEA) of these data showed that gene sets associated with oxidative phosphorylation (OXPHOS) and other mitochondrial-associated processes were changed in TE in SCe organoids compared to SCd, despite having no change at the RNA level (Fig. 1F and figs. S2, B and C). The opposite trend was observed in gene sets associated with glycolytic pathways, suggesting that this switch may be translationally regulated (Fig. 1F and figs. S2, B and C). When looking at specific genes, there are only minor changes to their levels. However, these changes are consistently present in many of the members of the gene sets, leading to the significant differences here observed. Previous studies have demonstrated that ISCs rely heavily on OXPHOS (*2*), and, in accordance with the RiboSeq results, we observed increased levels of mitochondrial respiration in SCe compared to SCd cultures (Fig. 1G and fig. S3B). When using various OXPHOS inhibitors to impair mitochondrial function (fig. S3C), we detected a significant decrease in the expression of stem cell markers (Fig. 1H), as quickly as within 15 min of treatment (fig. S3D), with only a slight impact on differentiation markers (fig. S3E). In contrast, we saw no consistent effect on the levels of these markers when using different glycolysis inhibitors (Fig. 1H and fig. S3E), supporting the idea that ISCs rely heavily on mitochondrial respiration. To measure whether this transcriptional change in ISC markers was indicative of a change in functional stem cells, we conducted a colony formation assay and observed that oligomycin treatment significantly impaired the clonogenic capacity of ISCs (Fig. 1, I to K), confirming that this is the case.

### NACα is translationally regulated in ISCs

**Fig. 2.**
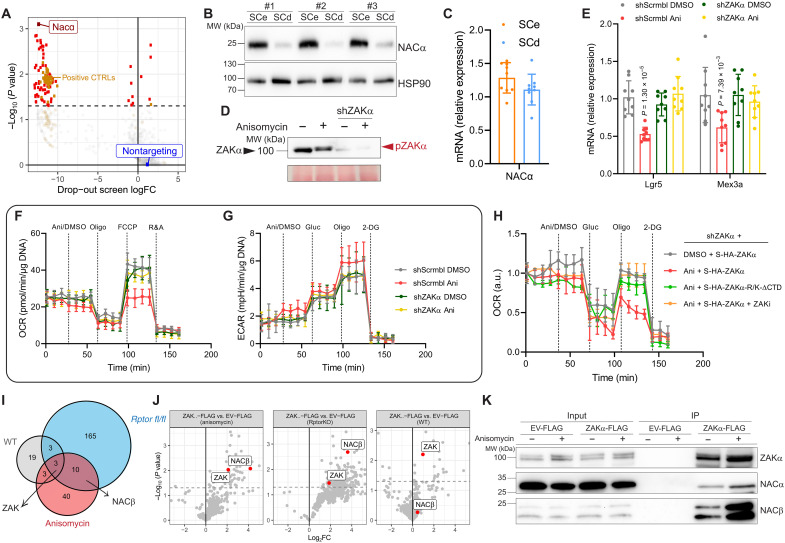
Intestinal cell regulation of NAC occurs posttranscriptionally. (**A**) Results of the CRISPR screen. Positive controls, nontargeting sgRNA and significant results are highlighted. Top hit is NACα (*n* = 3 biological replicates). (**B**) Western blot analysis shows higher levels of NACα in SCe compared to SCd cultures. HSP90 works as loading control (*n* = 3 biological replicates). (**C**) RT-qPCR analysis shows no differences in *NACα* levels in SCe compared to SCd cultures. *Hprt* works as a housekeeping control. Means and SD are shown (*n* = 3 biological replicates each in technical triplicates). (**D**) Phos-tag analysis shows ZAKα phosphorylation following anisomycin treatment. (*n* = 3 biological replicates). (**E**) RT-qPCR analysis of stem-related genes in WT organoids treated with anisomycin. *Hprt* works as a housekeeping control. Means and SD are shown (*n* = 3 biological replicates each in technical triplicates). (**F**) OCR analysis shows ZAKα-dependent decreased respiration in WT organoids treated with anisomycin. Means and SD are shown (*n* ≥ 4 biological replicates). Quantification in fig. S4C. (**G**) ECAR analysis shows ZAKα-dependent increased glycolysis WT organoids treated with anisomycin. Means and SD are shown (*n* ≥ 4 biological replicates). Quantification in fig. S4D. (**H**) OCR analysis shows rescue of the decrease in respiration, caused by anisomycin, by a ribosome-binding mutant version of ZAKα (S-HA-ZAKα-R/K-dCTD) and ZAKα inhibitor but not WT ZAKα (S-HA-ZAKα). Means and SD are shown (*n* ≥ 3 biological replicates). Quantification in fig. S4G. (**I**) Venn diagram of proteins identified in rapid immunoprecipitation mass spectrometry of endogenous protein (RIME) experiment as potential ZAKα interactors (*n* = 2 biological replicates). (**J**) Volcano plots highlighting differential enriched interactors of ZAKα (*n* = 2 biological replicates). (**K**) Immunoprecipitation of ZAKα-FLAG confirms interaction with both NACα and NACβ in HCT116 cells, which is increased upon anisomycin treatment. Empty vector (EV)-FLAG works as a control. (*n* = 3 biological replicates). Quantification in fig. S4I.

As we had shown that metabolism is translationally regulated in ISCs, we next assessed the functional importance of translationally regulated genes in ISCs. To do this, we performed a dropout CRISPR screen in SCe, Cas9-expressing organoids. We infected these organoids with a custom library containing single-guide RNAs (sgRNAs) targeting mRNAs that show altered translational levels in SCe and SCd organoids (table S2). The library contained a total of 4765 sgRNAs, of which 100 sgRNAs were nontargeting controls, 445 sgRNAs targeted genes known to be important for cell survival (such as ribosomal proteins), acting as positive controls, and 1235 sgRNAs were targeting translationally regulated genes involved in metabolic processes. Organoids were cultured in SCe medium for 10 days, and midway through this selection (day 5), the organoids were disrupted and recultured to promote stem cell capacity. Surviving organoids were harvested at day 10 and sequenced, allowing us to identify translationally regulated genes that are essential for stem cell capacity (fig. S4A). Alongside the positive controls, a total of 74 genes dropped out of the screen. As expected, there was no dropout of the nontargeting guides (Fig. 2A and table S3).

The top hit of the screen was the NAC subunit alpha, NACα (Fig. 2A and table S3). NACα is a subunit of the nascent polypeptide-associated complex (NAC), a highly conserved ribosome-associated complex whose primary function is to interact with and assist in the proper folding and targeting of newly synthesized polypeptide chains, as they emerge from the ribosome during translation (*18*). We confirmed that NACα levels were changed in SCe compared to SCd organoid cultures by Western blotting, which revealed a dramatic posttranscriptional down-regulation as ISCs differentiate (Fig. 2, B and C, and table S1). This finding suggests a potential role for NACα in ISCs maintenance, potentially via its regulation of metabolic identity.

### ZAKα regulates NACβ following translation impairment

We have previously shown that translation impairment also affects the metabolic identity of intestinal cells. This is regulated via the activation of the ribosome stress sensor ZAKα (MAP3K20), driving ISCs from OXPHOS toward glycolysis, which ultimately affects their identity in a p38-independent manner (*15*). However, how ZAKα mediates this metabolic switch is still unknown. To explore this, we first activated ZAKα in organoids using a low dose of a translation inhibitor (anisomycin) (Fig. 2D). This was sufficient to specifically decrease the expression of stem cell markers (Fig. 2E and fig. S4B) and rewire metabolism by decreasing OXPHOS levels (Fig. 2F and fig. S4C) and increasing glycolysis (Fig. 2G and fig. S4D), all of which were dependent on ZAKα, as demonstrated by the knockdown of the kinase using short hairpin RNA (shRNA). To confirm that the interaction of ZAKα with the ribosome was key in this context, we expressed either a mutant version of ZAKα that does not bind the ribosome (*24*) (S-HA-ZAKα-R/K-ΔCTD) or its wild-type (WT) ZAKα counterpart (S-HA-ZAKα) in shZAKα KD cells. As expected, mutant ZAKα cannot be activated upon anisomycin treatment (fig. S4E) and rescues the decrease in OXPHOS caused by anisomycin treatment (Fig. 2H and fig. S4F). Last, we also observed that targeting ZAKα kinase activity using a specific inhibitor (*25*) (fig. S4F) rescues the decrease in respiration triggered by anisomycin (Fig. 2H and fig. S4G), corroborating the findings seen upon genetic manipulation.

As ZAKα kinase activity is crucial in this context, it is likely that it regulates metabolism via direct interaction with a partner. We therefore generated an organoid line expressing a FLAG-tagged version of ZAKα. We treated these organoids with anisomycin, confirmed the activation of the FLAG-tagged ZAKα (fig. S4H), immunoprecipitated ZAKα, and carried out liquid chromatography–mass spectrometry (fig. S4I). We compared this to a similar experiment that we previously published (*15*), in which we used the same FLAG-ZAKα to identify interactors following impaired translation via mammalian target of rapamycin (mTOR) inhibition. Although both mTOR inhibition and anisomycin treatment activate ZAKα and inhibit OXPHOS, their interactome was unexpectedly different (Fig. 2I). Nevertheless, analysis of the overlapping interactors identified NACβ (also known as BTF3) as the most significantly enriched common ZAKα-interactor (Fig. 2, I and J). NACβ is the second subunit of the NAC, forming a heterodimer with NACα to assist in the cotranslational targeting of nascent polypeptides to the proper organelles.

As we had previously identified NACα as a potential regulator of mitochondrial metabolism in ISCs, this obviously piqued our interest. To confirm the interaction of ZAKα with the NAC, we used the colorectal cancer cell line HCT116, in which we overexpressed FLAG-ZAKα. We confirmed that NACβ interacts with ZAKα in these cells and that this interaction is increased upon anisomycin treatment. Furthermore, we also saw that NACα also interacts with ZAKα, albeit at a lower level than NACβ (Fig. 2K and fig. S4J).

### Altering NAC levels affects mitochondrial respiration and ISC identity

As we had identified NAC as a potential regulator of OXPHOS in two different model systems, we sought to further explore its role in metabolic regulation in these models and assess whether it plays a role in defining stem cell identity. Using intestinal organoids, we had observed a down-regulation of NACα upon cell differentiation (Fig. 2B), in which we also saw a reduction in OXPHOS (Fig. 1G and fig. S3B). We therefore hypothesized that overexpression of this gene would promote stemness by up-regulating OXPHOS.

**Fig. 3.**
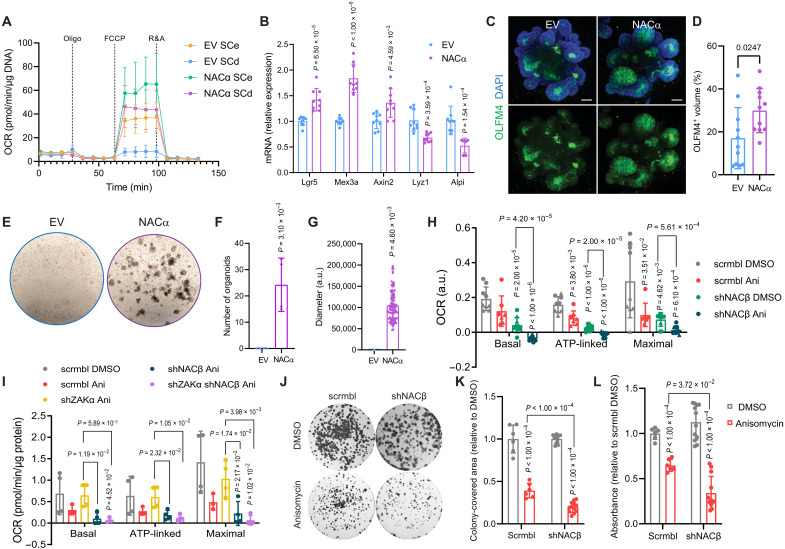
Modulation of NAC affects respiration and stemness in intestinal cells. (**A**) OCR analysis shows a rescue of respiration in SCd organoids overexpressing NACα. Means and SD are shown (*n* = 4 biological replicates). Quantification in fig. S5C. (**B**) RT-qPCR analysis of stem (*Lgr5*, *Mex3a*, and *Axin2*) and differentiation (*Lyz1* and *Alpi*) genes in organoids overexpressing NACα or EV, following R-spondin removal. *Hprt* works as a housekeeping control. Means and SD are shown (*n* = 3 biological replicates each in technical triplicates). (**C**) Immunofluorescence of organoids overexpressing NACα show a significant increase of stem cell marker OLFM4 (green) following R-spondin removal. DAPI (4′,6-diamidino-2-phenylindole, blue) marks the nuclei. Scale bar, 50 μM. (**D**) Quantification of stem cells based on the OLFM4^+^ signal in (C). (**E**) Representative images show that NACα overexpression significantly increases the clonogenic potential of SCd cultures (*n* = 4 biological replicates), measured by (**F**) number and (**G**) diameter of organoids. (**H**) OCR analysis shows decreased respiration in HCT116 cells treated with anisomycin, upon shNACβ and when combining both. Means and SD are shown [*n* = 5 biological replicates for each shRNA. shNACβ#3 (circles); shNACβ#1 (triangles)]. Profiles in fig. S5H. (**I**) OCR analysis shows decreased respiration in HCT116 cells treated with anisomycin and its rescue by shZAKα, which is reversed by shNACβ. Means and SD are shown (*n* = 4 biological replicates). Profiles in fig. S5I. (**J**) Representative images of survival assay in control and shNACβ HCT116 cells upon vehicle or anisomycin treatment. (**K**) Colony-covered area shows a decrease upon anisomycin treatment and a further reduction when combined with shNACβ. Means and SD are shown (*n* = 6 biological replicates). (**L**) Crystal violet shows a decrease upon anisomycin treatment and a further reduction when combined with shNACβ. Means and SD are shown (*n* = 6 biological replicates).

This turned out to be the case, and following NACα overexpression in WT organoids (fig. S5, A and B), we observed a substantial increase in mitochondrial respiration (Fig. 3A and fig. S5C). Moreover, when these organoids were grown in SCd media to promote differentiation, the expected decrease in OXPHOS was completely blocked, emphasizing the importance of NAC in this process (Fig. 3A and fig. S5C). NACα overexpression also resulted in an increase in stem cell markers and block of differentiation following R-spondin removal (Fig. 3B), demonstrating that NAC-mediated regulation of OXPHOS is key in sustaining the metabolic profile necessary for ISC maintenance. Direct visualization by immunofluorescence showed the presence of a significantly higher number of ISCs in NACα overexpressing organoids grown in SCd media, compared to WT organoids (Fig. 3, C and D). Furthermore, we tested the clonogenic capacity of ISCs in the same organoid lines and observed that NACα overexpression was sufficient to rescue colony formation capacity following R-spondin removal (Fig. 3, E to G), confirming that expression of NACα is sufficient to maintain ISCs in conditions that are known to cause their loss.

Alongside NACα overexpression, we also took the opposite approach and knocked down each subunit of NAC. As this was technically unfeasible in organoids, we used the HCT116 colon cancer cell line model. As expected, on the basis of the CRISPR screen results (Fig. 2A), NACα deletion was lethal in these cells. However, we successfully generated NACβ-deficient cells (fig. S5, D to F). These cells presented decreased OXPHOS, supporting NAC’s role as a regulator of metabolism (Fig. 3H and fig. S5H). Unexpectedly, when combining NACβ deficiency with anisomycin treatment [which results in a ZAKα-mediated decrease in OXPHOS (Fig. 3I and figs. S5, G and I)], there was an additive effect. We hypothesize that this is a result of incomplete knockdown of NACβ (figs. S5, D to F) and that the remaining protein is then fully inhibited by the activation of ZAKα, although it could also be explained by anisomycin and NACβ working in parallel pathways. As expected, anisomycin-mediated inhibition of OXPHOS was rescued by ZAKα knockdown (Fig. 3I and fig. S5I). Crucially, this rescue was completely abolished in NACβ-deficient cells, suggesting that the effect of ZAKα activation on OXPHOS is mediated by NACβ. Consistent with this, the loss of NACβ also exacerbated the effect of ribosome impairment on the survival of these cells. Using a colony formation assay, we observed that loss of NACβ further decreased both the colony-covered area and the crystal violet staining upon anisomycin treatment, supporting the idea that NAC deregulation decreases OXPHOS and proliferative potential of intestinal cells (Fig. 3, J to L). Together these results point to the importance of NAC in maintaining the metabolic profile necessary for ISC maintenance, demonstrating that NAC-regulated mitochondrial function plays a crucial regulatory role.

### Mitochondrial protein import is mediated by NAC via ribosome localization

**Fig. 4.**
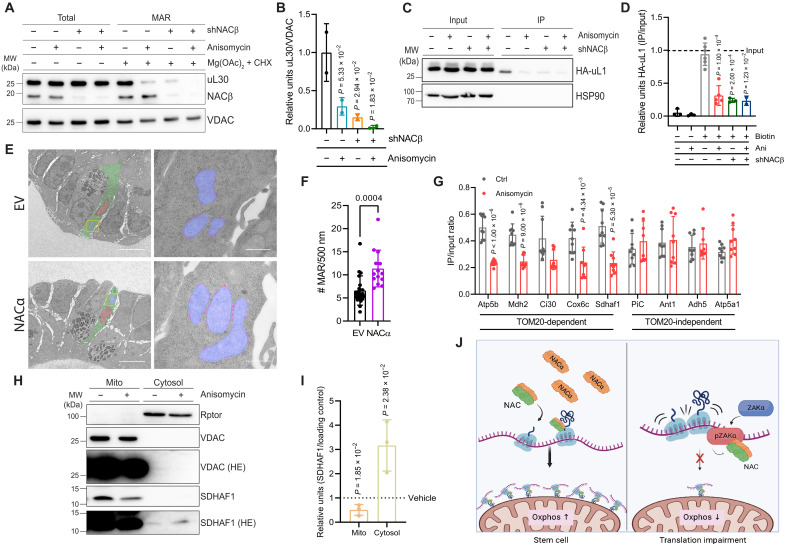
NAC promotes the import of mitochondrial proteins in intestinal cells via ribosome localization. (**A**) Western blot of HCT116 cells shows decrease in mitochondria-associated ribosomes (MAR) in shNACβ cells and upon anisomycin treatment. VDAC works as loading control. (**B**) Quantification of Western blots in (A). Means and SD are shown (*n* = 2 biological replicates). (**C**) Western blot of human embryonic kidney 293 (HEK293T) cells shows a decrease in biotinylated ribosomes following anisomycin treatment and shNACβ. HSP90 works as loading control. (**D**) Quantification of Western blots in (C). Means and SD are shown (*n* ≥ 2 biological replicates). (**E**) Representative electron microscopy (EM) images of WT organoids overexpressing EV-FLAG or NACα-FLAG. ISCs are marked in green (cytosol) and red (nulcei), and yellow squares indicate the matching zoomed-in areas. Scale bar, 50 μM. Zoomed-in images of the mitochondria (in blue) of ISCs show a significant increase MAR (in magenta) upon NACα overexpression. Scale bar, 500 nM. (**F**) Quantification of MAR in WT organoids overexpressing EV-FLAG and NACα-FLAG. Means and SD are shown, corresponding to ≥2 mitochondria from ≥5 images. (**G**) RT-qPCR analysis of genes with a putative N-terminal mitochondria-targeting sequence (MTS) (*Atp5b*, *Mdh2*, *Ci30*, *Cox6c*, and *Sdhaf1*) and without (*PiC*, *Ant1*, *Adh5*, and *Atp5a1*). *18S* rRNA works as housekeeping control. Means and SD are shown (*n* = 3 biological replicates each in technical triplicates). (**H**) Western blot of fractionated HCT116 cells shows decrease in SDHAF1 levels in mitochondria upon anisomycin treatment. Rptor and VDAC work as loading controls for cytosolic and mitochondrial fractions, respectively. (**I**) Quantification of Western blots in (H). Means and SD are shown (*n* = 3 biological replicates). (**J**) Schematic representation of the model proposed for the central role of NAC in metabolic regulation in the intestine. Created with BioRender.com.

It has been previously shown that NAC can regulate the localization of ribosomes (*19*, *20*). As the local translation of mitochondrial proteins is key to the correct functioning of this organelle (*26*), we reasoned that this may be an explanation of the observed phenotypes. To test this hypothesis, we used a fractionation-based method to measure the abundance of cytosolic ribosomes on the OMM (*27*), both in WT and NACβ-deficient cells, in the presence or absence of anisomycin. This analysis showed a significant decrease in the number of cytosolic ribosomes associated with the OMM following either anisomycin treatment or NACβ depletion, as measured by ribosomal protein uL30 levels (Fig. 4, A and B).

Although this finding supports the idea that impairment of NAC perturbs the localization of ribosomes to the OMM, this experiment relies on the addition of different drugs required to stabilize ribosomes, which may confound the interpretation of these results. To overcome this, we also performed a proximity-labeling assay using WT and NACβ-deficient cells expressing the OMM protein TOM20 fused to the biotin ligase BirA, which recognizes a specific avidin acceptor fused to the HA-tagged ribosomal protein uL1 (HA-uL1-AviTag) (fig. S6A) (*28*). After adding biotin to the cells, we immunoprecipitated the biotin-labeled avidin-tagged ribosomes using streptavidin beads and quantified the mitochondrially associated ribosomes (MARs) by determining the ratio of HA-uL1 levels in IPs-to-Inputs. This confirmed that following anisomycin treatment and consequent ZAKα activation, ribosomes were less localized to the mitochondria, an effect that was also seen following NACβ knockdown (Fig. 4, C and D). As NAC also blocks the mislocalization of proteins to the ER by preventing the signal recognition particle (SRP) from erroneously targeting ribosomes without a signal sequence (*19*), we also measured the distribution of ribosomes to the ER. We used the same system as above but using cells expressing HA-uL1-AviTag together with Sec63-BirA (*29*). This showed an increase in ER localized ribosomes following anisomycin treatment (figs. S6, B and C).

To visualize whether these same ribosome localization changes were occurring in the organoids, we used electron microscopy (EM) to measure MAR both in WT and following NACα overexpression. This analysis showed that NACα overexpression resulted in a significant increase in MAR (~30%) without any change in overall cytosolic ribosome levels (Fig. 4, E and F, and fig. S6D). Together, these results indicate that NAC regulates the distribution of ribosomes inside the cell, mediating their targeting to the mitochondria and potentially regulating OXPHOS.

To further understand what newly synthesized peptides might be affected by this MAR relocalization, we isolated the mRNAs associated with biotinylated ribosomes. As it is known that NAC probes the ribosomal exit tunnel for mitochondrial targeting sequence of nascent chains (*18*), which are then imported by TOM20 (*30*), we quantified mRNAs that encode for proteins that are thought to be imported by TOM20 (*Atp5b*, *Mdh2*, *Ci30*, *Cox6c*, and *Sdhaf1*) compared to mRNAs for mitochondrial proteins that are not thought to depend on TOM20 for their internalization to the mitochondria (*PiC*, *Ant1*, *Adh5*, and *Atp5a1*) (*28*). This revealed a significant decrease in the abundance of TOM20-dependent targets near the OMM, upon anisomycin treatment, while transcripts imported by other proteins were unaffected (Fig. 4G). Furthermore, through cell fractionation, we analyzed the protein levels of SDHAF1 in the mitochondria and in the cytosolic fraction. This TOM20-imported peptide is a component of the mitochondrial respiratory chain, and its translation in the vicinity of the mitochondria is reduced upon translation impairment and consequent NAC modulation (Fig. 4G). Consistent with this, we observed that upon anisomycin treatment, the amount of SDHAF1 in mitochondria decreases, and this is accompanied by an increase in the cytoplasm (Fig. 4, H and I). Together, these results suggest that NAC regulates the localization of ribosomes to the OMM and the consequent import of mitochondrial peptides through TOM20, thus potentially affecting mitochondrial metabolism and ultimately affecting cell identity in the intestine.

## DISCUSSION

The intestinal epithelium is the primary nutrient sensing organ in the body. It is in direct contact with nutrients and metabolites in the intestinal lumen and thus needs to constantly adapt to the current conditions by promoting a suitable differentiation program. During these transitions, different intestinal cells display specific metabolic programs; however, little is known about how such features are regulated. In this work, we explore how metabolism is regulated in intestinal cells.

It has previously been shown that mitochondrial quality control systems are important for this process, with mitochondrial fission (*14*) and stress response (*13*) playing crucial roles in intestinal stemness. However, our results suggest that this is only part of the picture. We show that ribosomes can modulate mitochondrial function by influencing localized translation at this organelle. Studies have demonstrated a role for localized translation in several contexts; however, these have exclusively focused on the spatial distribution of mRNAs (*31*, *32*). In this study, we show that the distribution of ribosomes is regulated in ISCs and that their redistribution may be an important part of both normal cell differentiation and the response to ribosome stress. While it is known that brain cells have the capability to reposition ribosomes during developmental processes and in response to injury (*33*, *34*), our study suggests that ribosome localization itself is a key determinant of cell fate.

We show that this process is mediated by NAC, which is regulated by translation during normal ISC function, and by the kinase Zakα following ribosome stress. In both contexts, the redistribution of ribosomes potentially acts as a regulator of metabolism, and NAC ultimately plays a crucial role in defining cell fate. (Fig. 4J). A potential explanation is that by promoting the relocalization of ribosomes to the outside of the mitochondria, NAC ensures that the metabolic needs of ISCs are met. Further studies are needed to specifically address the causative effect of ribosome localization on cellular metabolism, but this is technically impossible at present. However, if true, our study would bring phenotypic context to the still poorly understood role of NAC-mediated mitochondrial targeting of newly synthetized peptides and highlight the fact that this complex has a broader role beyond the regulation of SRP recognition of ER signal sequence–containing peptides (*18*, *35*).

Our findings also shed light on the intricate relationship between translation, metabolism, and stem cell function. As intestinal cells exhibit a high metabolic plasticity, switching between glycolysis and OXPHOS depending on their specific differentiation needs, the localization of ribosomes to the mitochondrial surface could indicate a specialized translational control mechanism that allows cells to rapidly adapt their protein synthesis in response to different demands. This flexibility is vital for cells to respond to environmental cues and maintain their functions under varying conditions, such as stress-related responses, which happen often in the intestine. Together, we show that the intersection of metabolic cues and signaling pathways determine the fate of ISCs in this complex tissue and that modulation of RNA translation via ribosome localization might be a key mediator of these processes.

It is worth mentioning that while the complexity of the organoid model is a major strength over standard cell line models, it does present some technical constraints. For example, in cell lines, we could measure ribosome localization, mRNA ribosome loading and protein import using both a BirA-proximity and a fractionation-based approach, which was impossible to do in organoids. As a result, we instead used EM, which allowed us to quantify MAR, but we could not assess ribosome loading or protein import as we have done in the cell lines. Nevertheless, the convergence of key phenotypic outcomes between these two distinct experimental approaches lends credibility to our results.

We also show that the metabolic program of ISCs is determined by translation rather than transcription, revealing a layer of regulation of metabolism in ISCs. This is key to allowing rapid responses that support the metabolic shifts required for self-renewal and/or differentiation into specific cell types. We used RiboSeq to identify this translational regulation and developed a metric to analyze these data. This was based not only on ribosomal abundance but also on the distribution of ribosomes along each transcript, a measure we called the RO s2b. This measure provides additional insights into translation dynamics, allowing us to identify mRNAs that have previously been missed by regular RiboSeq analysis. This includes *NAC*α, which had a clear difference in RO s2b, but not in overall RO, although its protein expression levels were significantly increased in ISCs. This highlights the power of using different readouts to get a more complete understanding of the translational regulation landscape. The combination of this with a custom-designed CRISPR screen that was also carried out in intestinal organoids provided a thorough and reliable analysis of the translational regulation of metabolism.

Our results revealed that ISCs produce high levels of NACα, thus favoring OXPHOS and maintaining their stem cell identity. However, upon either cell differentiation or translational impairment, NAC gets hindered, preventing the targeting of proteins to the vicinity of the mitochondria. This results in a decrease of cellular OXPHOS and changes in cellular phenotype. Consistent with this, overexpression of NACα is sufficient to rescue the decrease in mitochondrial respiration associated with differentiation, leading to an increase in functional ISCs. Together, these results highlight the complex regulatory roles that translation has in both the metabolism and the identity of intestinal cells. Here, we show that the intestine is capable of shaping its composition in a quick and adaptable manner, potentially by spatially regulating translation of mitochondrial proteins, essential to promote the survival of stem cells. We show that two distinct signals converge on NAC to regulate metabolism, positioning it as a central regulator of this process in the intestine. Overall, our findings emphasize the value of considering translation, and particularly ribosome dynamics, in the broader framework of cellular processes, and highlight the importance of the ribosome in understanding the intricate regulatory mechanisms that govern cell identity and function in the intestine.

## METHODS

### Ethical approval

All mouse experiments were carried out with the approval of the NKI Animal Welfare Body and the NKI-AVL Institutional Review Board, according to the ethical and procedural guidelines established by Dutch law.

### Mouse colonies

Experiments in this study included female and male C57BL/6 mice with ages between 8 and 12 weeks old, all bred in-house at the Netherlands Cancer Institute. The animals were generated as described previously (*15*). For *VillinCre^ERT2^eL22.HA* mice, two consecutive injections of tamoxifen (80 mg/kg) were administered, and samples were taken 24 hours after to adjust for variations in recombination efficiency and total cell count. Crypt cultures from *VillinCre^ERT2^Rptor^fl/f^* mice were induced in vitro, as described below. Gt(ROSA)26Sor^tm1.1(CAG-cas9*,-EGFP)Fezh^ mice did not require any induction.

### Organoid isolation and culture

Small intestinal organoids were generated from intestinal crypts of *VillinCre^ERT2^eL22.HA*, *VillinCre^ERT2^Rptor^fl/fl^*, and Gt(ROSA)26Sor^tm1.1(CAG-cas9*,-EGFP)Fezh^ mice, as previously described (*36*). Organoids were cultured in basal membrane extract (BME) (Amsbio) plugs in ENR medium: advanced Dulbecco’s modified Eagle’s medium (DMEM)/F12 (Thermo Fisher Scientific), supplemented with 10 mM Hepes (Thermo Fisher Scientific), 1× GlutaMAX (Thermo Fisher Scientific), penicillin (100 U/ml), streptomycin (100 μg/ml) (Thermo Fisher Scientific), 0.1% bovine serum albumin (Sigma-Aldrich), 1× B27 (Thermo Fisher Scientific), and 1× N2 (Thermo Fisher Scientific), together with 10% (v/v) Noggin-conditioned medium, 10% (v/v) R-spondin conditioned medium, and epidermal growth factor (50 ng/ml; Peprotech). All organoid lines were kept at 37°C in a humidified atmosphere with 5% CO_2_. For organoids isolated from *VillinCre^ERT2^Rptor^fl/fl^* animals, *Rptor* loss was induced in vitro by treatment with 500 nM 4-hydroxytamoxifen (Sigma-Aldrich).

SCe were generated by culturing organoids in ENR medium supplemented with 10 μM CHIR99021 (Cayman) and 1.5 mM of valproic acid (VPA, Biovision) (*22*) for 4 to 5 days. SCd cultures were obtained when culturing organoids in EN medium (the same media described above but depleted of R-spondin) for 2 days.

To inhibit OXPHOS, the organoids were treated for 24 hours with 2,5 μM oligomycin A, 1 μM rotenone, or 1 μM antimycin A. For glycolysis inhibition, the organoids were treated for 24 hours with 0.5 mM diclofenac or 15 mM dichloroacetate. All drugs used in thea Seahorse analysis were purchased from Sigma-Aldrich.

### Cell culture

HCT116 and human embryonic kidney (HEK) 293T were obtained from the American Type Culture Collection. HEK293T HA-uL1-AviTag and HA-uL1-AviTag Sec63-mVenus-BirA cells were a gift from J. S. Weissman’s laboratory (UCSF) (*29*) and Y. Arava’s laboratory (Technion) (*28*). All cells were cultured according to standard methods in DMEM high-glucose, Glutamax-supplemented medium (Thermo Fisher Scientific) supplemented with 10% fetal calf serum (FCS), penicillin (100 U/ml), and streptomycin (100 μg/ml) (Thermo Fisher Scientific), at 37°C in a humidified atmosphere with 5% CO_2_. All cell lines used were routinely tested for mycoplasma contamination.

### Plasmids, lentiviral production, and infection

LentiGuide-Puro (Addgene) (*37*) vector was used for cloning the custom CRISPR library used in the screen. shRNAs constructs for knockdown of Zakα in mouse intestinal organoids (target sequence: CCACGATTATCTGAACCTGTT) and NACβ in the human cell lines (target sequences: sh#3 CCCAGCATCTTAAACCAGCTT and sh#1 GCAGCGAACACTTTCACCATT) were chosen from the Open Biosystems Expression Arrest TRC library. As a negative control, shRNA containing a scramble sequence (ACAAGATGAAGAGCACCA) was used. These shRNAs were inserted into a pLKO.1 vector (Addgene). Constructs carrying either the ribosome-binding mutant version (S-HA-ZAKα-R/K-∆CTD) or WT Zakα (S-HA-ZAKα) were a gift from S. Bekker-Jensen’s group (University of Copenhagen) (*24*).

Lentivirus was produced by transfecting the above-mentioned vectors into HEK293T cells with the third-generation lentiviral packaging plasmids pVSV-G, pRSV-REV, and pMDL RRE (Addgene). Polyethylenimine (PEI, Polysciences) was the transfection agent used for plasmid delivery (3 μl/DNA μg). The viral supernatant was filtered through a 0.45-μm filter and, in the case of organoid infection, was concentrated using the LentiX concentrator reagent (Takara).

For the knockdown of ZAKα in HCT116 cells the commercial construct pLV[shRNA]-Puro-U6 > hMAP3K20[shRNA#5] (VB221006-1160bjs, VectorBuilder) was used (target sequence GATGTGACATTCAACACTAAC). The scramble shRNA lentiviral control vector pLV[shRNA]-EGFP/Puro-U6 > Scramble_shRNA (VB010000-0009mxc, VectorBuilder) was used as a control. For the affinity purification and immunoprecipitation experiments, ZAKα-FLAG was expressed through the construct pLV[Exp]-Bsd-mPGK>mMap3k20[NM_023057.5]/3xFLAG (VB210329-1381j, VectorBuilder), and the empty FLAG vector LV[Exp]-Bsd-mPGK> {3xFLAG/Stuffer_300bp} (VB210329-1386ht, VectorBuilder) was used as a negative control. For NACα overexpression, the commercial construct pLV[Exp]-Bsd-CMV > mNaca[NM_013608.3]/FLAG (VB230413-1266frg, VectorBuilder) was used. These constructs were packaged into lentivirus by VectorBuilder. Detailed information can be retrieved on vectorbuilder.com using the identifiers.

Cells were infected with lentivirus using polybrene (8 μg/ml; Sigma-Aldrich) and were subsequently selected with puromycin (2 μg/ml; Thermo Fisher Scientific) or blasticidin (8 μg/ml; Thermo Fisher Scientific) depending on the infected vector.

In organoid infections, organoids were kept in enriched ENR medium: ENR medium supplemented with growth and stem cell–inducing factors 10 μM Rho kinase inhibitor Y-27632 (Cayman), 1 mM VPA (Biovision), 1 μM Jagged-1 (AnaSpec), and 6 μM CHIR99021 (Cayman), for 2 days. Organoids were then dissociated into single cells using 1× TryplE Express Enzyme solution (Thermo Fisher Scientific) supplemented with deoxyribonuclease (DNAse, 200 U/ml) for 12 min at 37°C or StemPro Accutase (Thermo Fisher Scientific) supplemented with 10 μM Y-27632 for 3 min at 37°C, in the case of *Rptor ^fl/fl^* organoids. The cells were plated over BME-coated wells in enriched ENR medium and infected with the concentrated virus using polybrene (8 μg/ml). 24 hours after infection, a layer of BME was put on top of these cells and the medium was refreshed. Then, the organoids were selected with either puromycin (2 μg/ml; Thermo Fisher Scientific) or blasticidin (8 μg/ml; Thermo Fisher Scientific) depending on the infected vector. Modified organoids were then cultured in BME plugs with ENR medium.

For proximity assays, plasmid containing Tom20-mVenus-BirA was a gift from the Y. Arava’ laboratory (Technion) (*28*). This construct was transiently expressed in cells using PEI (Polysciences) as a transfection agent (3 μl/DNA μg).

### Colony formation and survival assays

For colony formation assays WT, NACα overexpressing or empty vector–expressing mouse intestinal organoids were dissociated into single cells using 1× TryplE Express Enzyme solution (Thermo Fisher Scientific) supplememented with DNAse (200 U/ml) for 12 min at 37°C. The reaction was stopped by washing the dissociated cells with advanced DMEM/F12 (Thermo Fisher Scientific), supplemented with 10 mM Hepes (Thermo Fisher Scientific), 1× GlutaMAX (Thermo Fisher Scientific), penicillin (100 U/ml), and streptomycin (100 μg/ml) (Thermo Fisher Scientific). A total of 100,000 cells were seeded per 10 μl of BME plug. For WT organoids, the cells were cultured for 24 hours after seeding in ENR media supplemented with 10 μM Y-27632 and either 2.5 μM oligomycin A (Sigma-Aldrich) of dimethyl sulfoxide (DMSO). For NACα overexpressing and empty vector–expressing organoids, the cells were cultured for 24 hours after seeding in SCd conditions (EN medium supplemented with 10 μM Y-27632). For all lines, the organoids were then grown in ENR medium for five more days and images were acquired.

HCT116 cells were plated in six-well plates at around 1500 cells per well and cultured in DMEM, as described above, for 10 days. Upon plating, cells were either treated with anisomycin (1 μM, Sigma-Aldrich) or vehicle (DMSO) for 24 hours. After 10 days, the cells were fixed and stained with crystal violet solution [0,025% crystal violet (Sigma-Aldrich), 1% MetOH, and 1% formaldehyde] for 1 hour and then washed with water. Images were acquired in a ChemiDoc XRS+ (BioRad), and colony-covered area was quantified using Fiji software. Crystal violet staining was extracted by incubating plates with 10% acetic acid solution for 40 min, and 562-nm absorbance was measured using an Infinite M Plex microplate reader (Tecan).

### Immunofluorescence

Immunofluorescence of intestinal organoids was performed as previously described (*38*). SC-depleted organoids were incubated overnight with anti-OLFM4 antibody and Alexa Fluor–labeled secondary antibody was later combined with 4′,6-diamidino-2-phenylindole (DAPI). Images were collected on BC43 Benchtop spinning disk confocal (Andor, Oxford Instruments). A Plan-Apochromat 20×/0.8 WD = 0.8-mm air objective was used to obtain 16-bit images with a 4.1 MP complementary metal-oxide semiconductor camera with a 2-by-2 binning (resolution: 1024 by 1000). A *z*-slice thickness of 2 μM was used to cover the complete thickness of the sample (200 μM, 101 slices). Imaris software (version 10.1.0, Oxford Instruments) was used for three-dimensional reconstruction of images and their quantification. In short, the “surface” function was used to generate a mask of the total volume of the epithelium of each organoid (based on the DAPI channel). Within this volume, the surface function was used to measure the total ISC volume (based on the OLFM4 channel), thereby excluding the unspecific luminal signal. The OLFM4^+^ volume was calculated by dividing the sum of the volume of all OLFM4^+^ surfaces in one organoid over the total epithelial volume per organoid. A list of the antibodies used can be found in table S4.

### Ultrastructural analysis and quantification

Organoids were cultured as mentioned above and prepared for EM similar to as previously reported (*14*). Briefly, samples were fixed in Karnovsky fixative [2.5% glutaraldehyde (EMS) + 2% formaldehyde (Sigma-Aldrich)] in 0.1 M PHEM buffer [60 mM PIPES, 25 mM Hepes, 10 mM EGTA, and 2 mM MgCl_2_ (pH 6.9)] at room temperature for 2 hours. They were rinsed and stored in 1% formaldehyde 1 M PHEM buffer (pH 6.9) at 4°C until further processing. The samples were postfixed with 1% OsO_4_, 1.5% K_3_Fe(III)(CN)_6_ in 1 M PHEM (pH 7.4) for 2 hours. The organoids were then dehydrated in a degraded series of acetone and embedded in EMbed812 (EMS). Ultrathin sections were cut (Leica Ultracut UCT), collected on formvar transmission EM grids (Cell Microscopy Core, UMC Utrecht) after fresh carbon coating (Leica EM ACE600). Sections were post-stained with uranyl acetate and lead citrate (Leica AC20). Micrographs were collected on a Tecnai12 (FEI Thermo Fisher Scientific) equipped with a Veleta 2 k–by–2 k charge-coupled device (CCD) camera (EMSIS, Munster, Germany) operating SerialEM software or on a JEM1010 (JEOL) equipped with a Veleta 2 k–by–2 k CCD camera (EMSIS, Munster, Germany).

For quantifying the number of MAR, entire mitochondrial perimeters were selected using a freehand selection tool and the length was measured in FiJi (*39*). The ribosomes in contact (<20-nm proximity) with the selected mitochondrial membranes were annotated, and the number of ribosomes per measured mitochondrial membrane length (ribosome #/500 nm) was calculated per condition in randomly selected cells. In addition, the number of cytosolic ribosomes was quantified in the same cells by selecting random areas of 200 nm by 200 nm in the cytosol and calculating the number of ribosomes per measured cytosolic area (ribosome #/μm^2^). The annotations and quantifications of EM images were done by three independent people.

### RNA isolation and RT-qPCR

Cells were collected on ice, washed in phosphate-buffered saline (PBS), and centrifuged at 1000 rpm for 5 min at 4°C. The pellets were lysed with TRIzol reagent (Thermo Fisher Scientific). RNA was isolated by chloroform extraction followed by centrifugation, isopropanol precipitation, washing in 75% ethanol, and resuspension in nuclease-free water. Nucleic acid quantification was performed with Nanodrop, and 1 μg of template was used for downstream analysis. Reverse transcription reactions were carried out using the High-Capacity cDNA Reverse Transcription Kit (Thermo Fisher Scientific), following the manufacturer’s instructions. qPCR was performed using the comparative CT method by normalization of targets of interest to a suitable housekeeping gene (table S5). SYBR Green PCR Master Mix (Thermo Fisher Scientific) reactions were carried out in technical triplicates in a final volume of 7 μl.

### Protein synthesis assay

Protein synthesis rates were measured as described previously (*36*). Briefly, intestinal organoids were grown in either ENR, SCe, or SCd conditions and taken on day 4 for analysis. The cells were incubated with DMEM methionine-free medium (Thermo Fisher Scientific) for 20 min, after which ^35^*S*-methionine label (30 μCi/ml; Hartmann Analytic) was added for 1 hour. The cells were harvested in ice-cold PBS and centrifuged at 800 rpm for 3 min to clear the BME. Pellets were resuspended in lysis buffer [50 mM tris-HCl (pH 7.5), 150 mM NaCl, 1% Tween-20, 0.5% NP-40, 1× protease inhibitor cocktail (Roche), and phosphatase inhibitor cocktail (Sigma-Aldrich)] and incubated on ice for 10 min. The lysates were then cleared by centrifugation at 13,000 rpm for 2 min and precipitated onto filter paper (Whatmann) with 25% trichloroacetic acid and washed twice with 70% cold ethanol and twice with cold acetone. Last, a liquid scintillation counter (PerkinElmer) was used to measure scintillation, and the activity was normalized by total protein content. All experiments were done in technical replicates for each biological unit.

### Bioenergetics analysis

Assessment of the cellular metabolic function was carried out using Seahorse Bioscience XFe24 Analyzer (Agilent). Measurements in organoids were done as described previously by our group (*15*). For HCT116 cells, these were seeded at 25,000 cells per well and cultured in DMEM, as described above, for 48 hours before the analysis. Both oxygen consumption rate (OCR) (representing mitochondrial respiration) and extracellular acidification rate (ECAR) (representing glycolysis) measurements were taken according to the manufacturer’s instructions in DMEM (Sigma-Aldrich) supplemented with 2 mM l-glutamine for the ECAR experiments and additional 5.5 mM d-glucose for the OCR measurements. For OCR assays, the following reagents were added: oligomycin A (1 μM), FCCP (1 μM) (Sigma-Aldrich), rotenone (1 μM), and antimycin A (1 μM). For the ECAR analysis the following reagents were added: glucose (10 mM) (Sigma-Aldrich), oligomycin A (1 μM) (Sigma-Aldrich), and 2-deoxy-d-glucose (50 mM) (Sigma-Aldrich). When stated, the cells were pretreated with either ZAKi (1 μM) (*25*), anisomycin (1 μM, Sigma-Aldrich), or vehicle (DMSO) for 30 min. Results were normalized to DNA content for the organoids. Briefly, cells were dissolved in DNA lysis buffer [75 mM NaCl, 50 mM EDTA, 0.02% SDS, and Proteinase K (0.4 mg/ml)] and incubated at 56°C for 2 hours. DNA was then precipitated by mixing samples with 1 volume of isopropanol, followed by an incubation at 4°C overnight and centrifugation at 8000 rpm for 30 min at 4°C. Pellets were washed with cold 70% ethanol and air dried. Last, DNA was resuspended in nuclease-free H_2_O and quantified using Nanodrop. For cell lines, total protein content was used to normalize the results. Cells were lysed in radioimmunoprecipitation assay lysis buffer [50 mM tris-HCl (pH 8.0), 150 mM NaCl, 1% NP-40, 0.5% sodium deoxycholate, 0.1% SDS, and 1× protease inhibitor cocktail cOmplete ULTRA tablets, EDTA-free (Roche)], incubated for 10 min on ice and centrifuged for 20 min, max speed at 4°C. The supernatants were quantified using the Pierce bicinchoninic acid (BCA) Protein Assay Kit (Thermo Fisher Scientific) and an Infinite M Plex microplate reader (Tecan).

### Western blot

The organoids and cells were washed twice with cold PBS, and pellets were resuspended on ice in the appropriate lysis buffer. Samples were then sonicated for 10 cycles of 1 s ON/1 s OFF, with an amplitude between 20 and 30%. After quantifying proteins with the Pierce BCA Protein Assay Kit (Thermo Fisher Scientific) and an Infinite M Plex microplate reader (Tecan), these were separated by SDS–polyacrylamide gel electrophoresis (SDS-PAGE) and transferred to a 0.2-μm pore nitrocellulose membrane (PALL). Membranes were blocked using 5% milk/tris-buffered saline 0.1% Tween 20) and then incubated with primary antibodies overnight at 4°C followed by the corresponding secondary antibody conjugated to horseradish peroxidase for 1 hour at room temperature. Last, proteins were visualized with the help of ECL-Plus reagent (Thermo Fisher Scientific) and Syngene equipment. The antibodies used in this study can be found in table S4.

### Phos-tag gel electrophoresis

Cells were lysed in 2× NuPAGE LDS sample buffer (Thermo Fisher Scientific) with 100 μM dithiothreitol (DTT), sonicated, and boiled for 10 min at 100°C. The samples were run in 8% SDS-PAGE gels prepared with 10.7 μM Phos-tag acrylamide (Wako) and 21.4 μM MnCl_2_. Before transferring, the gel was rinsed three times with transfer buffer supplemented with 10 mM EDTA and then one final time with EDTA-free transfer buffer. Transfer and subsequent procedure was performed as described for Western blot.

### RNA sequencing

Total RNA was isolated from SCe and SCd organoid cultures as described above. The quality of the samples was assessed with the 2100 Bioanalyzer, using an RNA Nanochip (Agilent), and used in downstream analysis when showing an RNA integrity number above 8. Libraries were generated with the TrueSeq Stranded mRNA kit (Illumina) and sequenced using HiSeq2500 equipment.

### Ribosome profiling

#### Sample preparation

The samples were prepared as described previously (*15*). Briefly, intestinal organoids were generated from *VillinCre^ERT2^RPL22.HA* and *VillinCre^ERT2^Rptor^fl/fl^RPL22.HA* mice and plugged in 30 μl of BME. Each experimental condition was carried out using three biological replicates, and around 150 plugs were used for each replicate. Translating ribosomes were stalled by treating cells with cycloheximide (100 μg/ml) for 3 to 5 min at 37°C and immediately incubating them on ice for the remainder of the experiment. After collecting the cells, the pellets were washed twice with cold PBS supplemented with cycloheximide (100 μg/ml), resuspended in ice-cold lysis buffer [20 mM tris HCl (pH 7.4), 10 mM MgCl_2_, 150 mM KCl, 1% NP-40, cycloheximide (100 μg/ml), and 1× EDTA-free proteinase inhibitor cocktail (Roche)], and incubated for 20 min on ice, followed by mechanical disruption with a 25G syringe. Lysates were then centrifuged at max speed for 20 min at 4°C, and the supernatants were collected.

#### eL22-HA pull down

Lysates were precleared with Pierce Control Agarose Matrix (Thermo Fisher Scientific) for 20 min at 4°C and immunoprecipitated with prewashed Anti-HA.11 Epitope Tag Affinity Matrix (BioLegend) for 4 hours at 4°C. The beads were washed twice with ice-cold lysis buffer and twice with ice-cold wash buffer [20 mM tris HCl (pH 7.4), 10 mM MgCl_2_, 350 mM KCl, 1% NP-40, cycloheximide (100 μg/ml), and 1× EDTA-free protease inhibitor cocktail (Roche)]. Tagged ribosomes were then eluted by incubating the beads with HA peptide (200 μg/ml; Thermo Fisher Scientific) for 15 min at 30°C with constant agitation. Digestion of nonprotected RNA was performed with 10 μl of ribonuclease I (Thermo Fisher Scientific) for 40 min at 25°C, and the reaction was stopped by adding 13 μl of SUPERASE (Thermo Fisher Scientific). Lastly, RPFs were purified using miRNeasy minikit (Qiagen) following the manufacturer’s instructions.

#### Library preparation

The library preparation was performed as previously described (*15*). RPFs ranking from 19 to 32 nucleotides were size-selected using a 10% TBE-Urea polyacrylamide gel. The 3′ and 5′ ends were modified accordingly, and the respective adapters were ligated with a T4 RNA ligase I (New England Biolabs). The final products were size-selected one last time, and ribosomal RNA (rRNA) depletion was performed using custom-made biotinylated oligos (table S6) (*40*) together with MyOne Streptavidin C1 DynaBeads (Thermo Fisher Scientific). Purified RPFs were then used to synthesize cDNA with SuperScript III (Thermo Fisher Scientific), according to the manufacturer’s instructions, using the RTP primer (table S5). After purification with G50 columns (Merck), cDNA was amplified using Phusion High-Fidelity DNA Polymerase (Thermo Fisher Scientific), with primers containing different RP indexes to allow for sequencing (table S5). PCR products were purified with a QIAquick PCR purification kit (Qiagen) and size-selected one last time with an E-Gel SizeSelect II 2%, (Thermo Fisher Scientific). The quality and molarity of all samples were assessed with the Agilent 2100, and libraries were sequenced on the Illumina HiSeq2500.

#### RNA-seq and RiboSeq data analysis

All sequencing datasets were first quality controlled using the FastQC tool. Ribosome profiling data were later trimmed and cleaned from adapter sequences using the Cutadapt tool (*41*). RiboSeq-related QC plots were generated with RiboCode (*42*) after mapping the rRNA and tRNA–cleaned reads to mm10 genome using the STAR aligner (*43*). Transcript quantifications were performed with Salmon (v.1.1.0) (*44*), for which transcript sequences from gencode vM21 annotation were used. However, coding sequence (CDS) and untranslated region (UTR) sequences of each gene were separated for the quantification of RO with RiboSeq reads. Before transcript quantifications, the sequencing reads were also cleaned from rRNA fragments with the SortMeRNA tool (*45*). In subsequent analyses, upstream open reading frame (uORF) RO was defined as the RO levels determined on the 5′UTR sequences of each gene. Differential TE of each gene was calculated with the RiboDiff tool (*46*), for which the input consisted of salmon-computed RNA-seq–based mRNA abundance and RiboSeq-based CDS RO values for every primary transcript of each gene. Primary transcripts were determined by the APPRIS annotation, and low-abundance genes were excluded from this analysis. The other gene-specific translation metric, RO s2b ratio, was defined as the ratio of read counts between the translation initiation site (first 30 codons) and the rest of the CDS, where each gene is represented by its primary transcript. Note that this ratio is also multiplied by the CDSlength-90 to minimize the effect of varying CDS lengths on RPF abundance, and genes/transcripts with a low number of RPFs or CDS length smaller than 100 are excluded from the analysis. Differential analysis of RNA abundance, RO, and uORF RO were performed with the DESEQ2 package (*47*) in the R environment, whereas, limma package (*48*) was used for differential s2b ratio analysis. GSEA were performed with the ClusterProfiler package (*49*) using the standard GSEA measure that was defined as logFC × min[−log_10_(adj *P* value),3], except for the differential s2b ratio where standard *P* values were used instead of adjusted ones.

### CRISPR screen

#### Library design

To assess the essentiality of translationally regulated genes in ISCs, we created a customized CRISPR library where target genes/regions were selected on the basis of analysis-specific criteria and manual selection of control targets. From our analyses, we included all genes with significant TE changes (adj. *P* < 0.1) or s2b ratio changes (*P* < 0.1) to be targeted in the CDS region. Similarly, genes with significant RO change (adj. *P* < 0.05) but with no significant change in expression (adj. *P* > 0.05) were also targeted at the CDS region. Genes with significant uORF RO change (adj. *P* < 0.05) were targeted in three separate regions; CDS, uORF (UTR5), and upstream region from the uORF (UP). For all CDS regions, we selected five guides from Brie (*50*) and GeCKO (*51*) libraries, whereas, for each UTR5 and UP regions, we designed five highly efficient and specific guides with the help of CRISPRon (*51*) and CRISPRoff (*52*) web servers.

#### Custom library cloning

Custom library was ordered from Twist Biosciences. Esp3I recognition sites were appended to each sgRNA sequence together with appropriate overhangs sequences and adaptor identifiers to allow differential amplification from the ordered synthesis pool by PCR with primers *PC1* (table S5). The PCR product was purified using the MinElute PCR Purification kit (Qiagen). Amplicons were cloned into the LentiGuide-Puro vector (digested according to the lentiviral CRISPR toolbox protocol developed by Zhang laboratory (*37*) (Addgene’s website) and purified by agarose gel electrophoresis using the Wizard SV Gel and PCR Clean-Up System (Promega) via Golden Gate cloning with FastDigest Esp3I (Thermo Fisher Scientific) and T7 DNA ligase (3000 U/μl) (New England Biolabs). The product was purified and transformed into Endura DUOs Electrocompetent cells (LGC, Biosearch Technologies) according to the manufacturer’s instructions. DNA was purified using the PureLink HiPure Plasmid Filter Maxiprep kit (Invitrogen), and equal sgRNA coverage within the library was confirmed by sequencing.

#### Library transduction and selection

Lentivirus was produced, and Cas9-expressing organoids were transduced at 600-fold complexity of the library at a multiplicity of infection of 0.3 for each of the three technical replicates. The precise working virus titer was determined in advance in a viral titration experiment on non–Cas9-expressing organoids in which survival was measured following puromycin selection. Cells (60 × 10^6^) were plated on a BME layer, infected with the determined viral volume and 24 hours after transduction their growth into organoids was promoted by overlaying them with BME. A day after this puromycin selection started (2 μg/ml) and after 48 hours, selection of infected organoids was completed and T0 triplicates were collected. The replicates of the experimental arm were cultured for 10 days in ENR medium supplemented with 10 μM CHIR99021 (Cayman) and 1.5 mM of VPA (Biovision). On day 5 organoids were harshly disrupted by pipetting to potentiate their stem capacity. The surviving organoids were collected on day 10.

#### Barcode amplification and sequencing

DNA of all time points and replicates was extracted using the DNeasy Blood and Tissue kit (Qiagen). sgRNA cassettes were amplified from the collected genomic DNA, and Illumina sequencing adaptors and sample barcodes were introduced in two consecutive PCR reactions, first with primers *PCR1* and then with *PCR2* primers (table S5), using Phusion polymerase (2 U/μl) (Thermo Fisher Scientific). PCR products were purified with a QIAquick PCR Purification kit (Qiagen), molarity was assessed with the Agilent 2100, and samples were sequenced on the Illumina NextSeq550.

#### Data analysis

Adapter trimming of sequencing data was performed with cutadapt (*41*). Quality control and drop-out analysis was performed with MAGECK-VISPR pipeline (*53*).

### Affinity purification and quantitative mass spectrometry

Rapid immunoprecipitation mass spectrometry of endogenous proteins (RIME) was used to identify potential ZAKα interactors as previously described (*15*, *54*). In addition to the WT and *Rptor^fl/fl^* organoids used in our previous work (*15*), we also included one condition of WT organoids treated with 1 μM of anisomycin for 30 min. The exact same conditions were used to immunoprecipitate ZAKα from HCT116 cells, with Western blot analysis being the main readout to test interaction partners.

### MAR isolation

MAR samples were isolated as previously described (*27*) with a few alterations. Briefly, cells were treated with cycloheximide (50 μg/ml) and 2 mM Mg(OAc)_2_ upon either anisomycin (1 μM) or vehicle (DMSO) treatment for 30 min. The cells were then harvested in extraction buffer [0.25 M sucrose, 20 mM Hepes-KOH (pH 7.5), 10 mM KCl, 1.5 mM MgCl_2_, 1 mM EDTA, 1 mM EGTA, 1 mM DTT, 0.1 mM phenylmethylsulfonyl fluoride, cycloheximide (50 μg/ml), and 2 mM Mg(OAc)_2_] and homogenized on ice using a Teflon glass apparatus (loose pestle) 20 times. After centrifugation at 750*g* for 10 min at 4°C, the supernatants were collected and the pellets were resuspended in an extraction buffer and once again homogenized and centrifuged with the same settings. Both supernatants were combined and centrifuged at 10,000*g* for 15 min at 4°C. Pellets containing crude mitochondria were resuspended in sucrose/MOPS buffer [250 mM sucrose, 10 mM MOPS-KOH (pH 7.2), cycloheximide (50 μg/ml), and 2 mM Mg(OAc)_2_], and proteins were solubilized in Laemmli buffer with 50 mM DTT, at 65°C for 15 min, and analyzed by Western blot. A list of the antibodies used can be found in table S4.

### Proximity assays

HEK293T cells expressing HA-uL1-AviTag, Tom20-mVenus-BirA, and/or Sec63-mVenus-BirA were grown in DMEM (Thermo Fisher Scientific) supplemented with 10% (v/v) dialyzed FCS and penicillin (50 U/ml)/streptomycin (50 μg/ml). Cells were treated with cycloheximide (100 μg/ml; Sigma-Aldrich) for 2 min at 37°C, followed by labeling with D-biotin (50 μM) (Sigma-Aldrich) for 20 min at 37°C. After washing with PBS supplemented with cycloheximide (100 μg/ml), the cells were collected by scraping in lysis buffer [20 mM tris (pH 7.5), 150 mM NaCl, 5 mM MgCl_2_, 2% Triton X-100, 1 mM DTT, and cycloheximide (100 μg/ml)] and lysed on ice for 5 min. Lysates were then cleared by centrifugation at 3000*g* for 15 min at 4°C, and the supernatants were loaded on a Zeba desalt spin column (Thermo Fisher Scientific). Proteins were quantified using the Pierce BCA Protein Assay Kit (Thermo Fisher Scientific), and 100 μl were saved for input control. Two milligrams of total protein extracts was incubated with 50 μl of prewashed Dynabeads MyOne Streptavidin C1 beads (Thermo Fisher Scientific) for 1 hour at 4°C using an overhead tumbler. The supernatant was removed with the help of a magnetic rack, and the beads were washed three times with a high-salt wash buffer [20 mM tris (pH 8.0), 750 mM KCl, 5 mM MgCl_2_, 0.1% Triton X-100, 0.5 mM DTT, and cycloheximide (100 μg/ml)] for 15 min at 4°C. Biotinylated proteins were eluted from the beads with 10 U of TEV protease (Invitrogen) according to the manufacturer’s instructions. For Western blot analysis, 10% of input and 25 μl of Strep-IP elute were analyzed by Western blot as described above. For RT-qPCR analysis, the protocol was the same as described, but the elution was done by adding TRIzol to the beads and proceeding with the RNA isolation, cDNA synthesis and real-time qPCR, as described above, using the primers listed in the table S5.

### Cell fractionation

Cells were lysed in STM buffer [250 mM sucrose, 50 mM tris-HCl (pH7.4), and 5 mM MgCl_2_] on ice for 30 min. After centrifugation at 4°C, 800*g* for 15 min, the supernatants were collected and centrifuged again for 10 min. The supernatants were once more centrifuged at 4°C for 10 min, this time at 11,000*g*, the pellets and supernatants were collected for mitochondria and cytosolic isolation, respectively. Pellets were resuspended in 200 μl of STM buffer and centrifuged at 11,000*g* for 10 min, 4°C. The resulting pellets were resuspended in 100 μl of SOL buffer [50 mM tris-HCl (pH 6.8), 1 mM EDTA, and 0.5% Triton X-100] and sonicated three times for 10 s, avoiding warm-up. For the cytosolic fractions, the supernatants were precipitated in acetone for 1 hour at −20°C and centrifuged at 4°C, 12,000*g* for 5 min. Pellets were resuspended in 300 μl of STM buffer.

### Statistical analyses

For each experiment, the number of replicates is indicated in the figure legends, and *P* values were determined using a two-tailed *t* test.
