# Supplementary material for: NAC regulates metabolism and cell fate in intestinal stem cells
Source: Sci Adv. 2025 Jan 8;11(2):eadn9750. doi: 10.1126/sciadv.adn9750 (PMC11708876; doi:10.1126/sciadv.adn9750)
Supplement: Supplementary file 1 — Figs. S1 to S6 Legends for tables S1 to S6 [file sciadv.adn9750_sm.pdf]

Science Advances
AAAS

Supplementary Materials for

NAC regulates metabolism and cell fate in intestinal stem cells

Sofia Ramalho *et al.*

Corresponding author: William J. Faller, w.faller@nki.nl; Joana Silva, j.silva@nki.nl

Sci. Adv. **11**, eadn9750 (2025)
DOI: 10.1126/sciadv.adn9750

The PDF file includes:

Figs. S1 to S6
Legends for tables S1 to S6

Other Supplementary Material for this manuscript includes the following:

Tables S1 to S6

Fig. S1

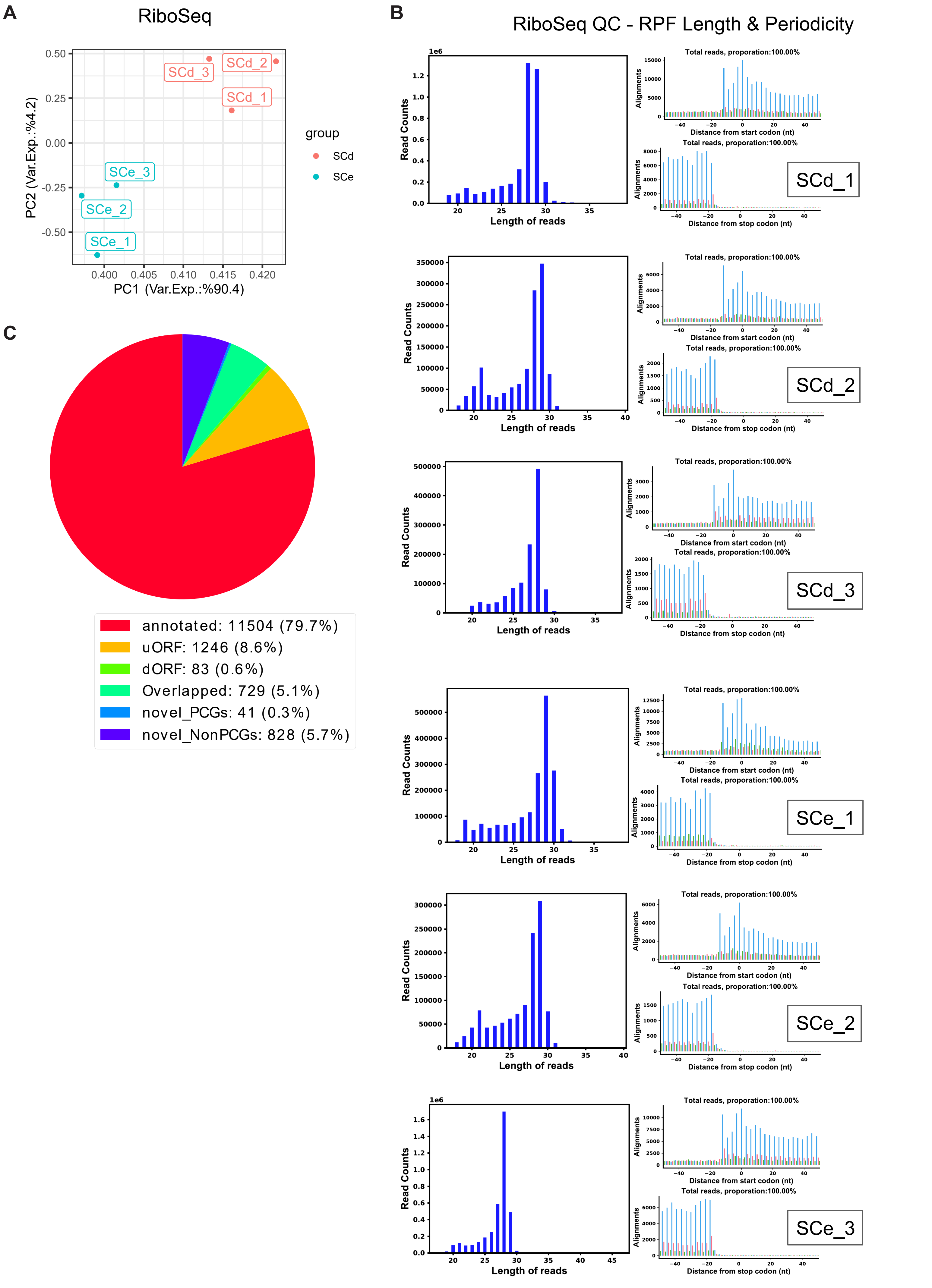

A
RiboSeq
PC1 (Var.Exp.:%90.4)
PC2 (Var.Exp.:%4.2)
group
SCd
SCe
SCd_1
SCd_2
SCd_3
SCe_1
SCe_2
SCe_3
B
RiboSeq QC - RPF Length & Periodicity
Read Counts
Length of reads
Total reads, proporation:100.00%
Alignments
Distance from start codon (nt)
Distance from stop codon (nt)
SCd_1
SCd_2
SCd_3
SCe_1
SCe_2
SCe_3
C
annotated: 11504 (79.7%)
uORF: 1246 (8.6%)
dORF: 83 (0.6%)
Overlapped: 729 (5.1%)
novel_PCGs: 41 (0.3%)
novel_NonPCGs: 828 (5.7%)

Fig. S2

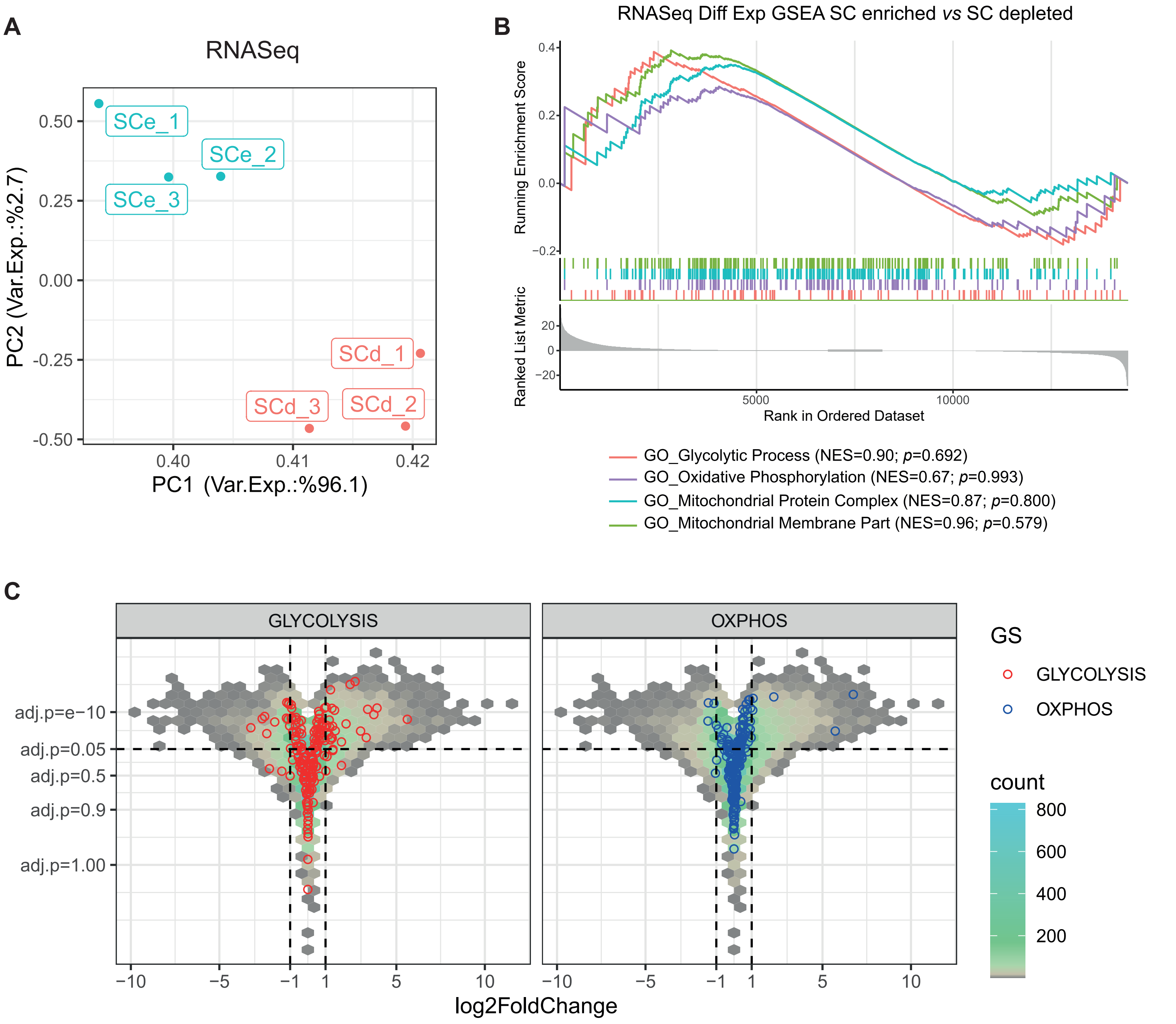
A
RNASeq
SCe_1
SCe_2
SCe_3
SCd_1
SCd_2
SCd_3
PC2 (Var.Exp.:%2.7)
PC1 (Var.Exp.:%96.1)
0.50
0.25
0.00
-0.25
-0.50
0.40
0.41
0.42
B
RNASeq Diff Exp GSEA SC enriched vs SC depleted
Running Enrichment Score
Ranked List Metric
Rank in Ordered Dataset
5000
10000
GO_Glycolytic Process (NES=0.90; p=0.692)
GO_Oxidative Phosphorylation (NES=0.67; p=0.993)
GO_Mitochondrial Protein Complex (NES=0.87; p=0.800)
GO_Mitochondrial Membrane Part (NES=0.96; p=0.579)
C
GLYCOLYSIS
OXPHOS
adj.p=e−10
adj.p=0.05
adj.p=0.5
adj.p=0.9
adj.p=1.00
log2FoldChange
GS
GLYCOLYSIS
OXPHOS
count
800
600
400
200

Fig. S3

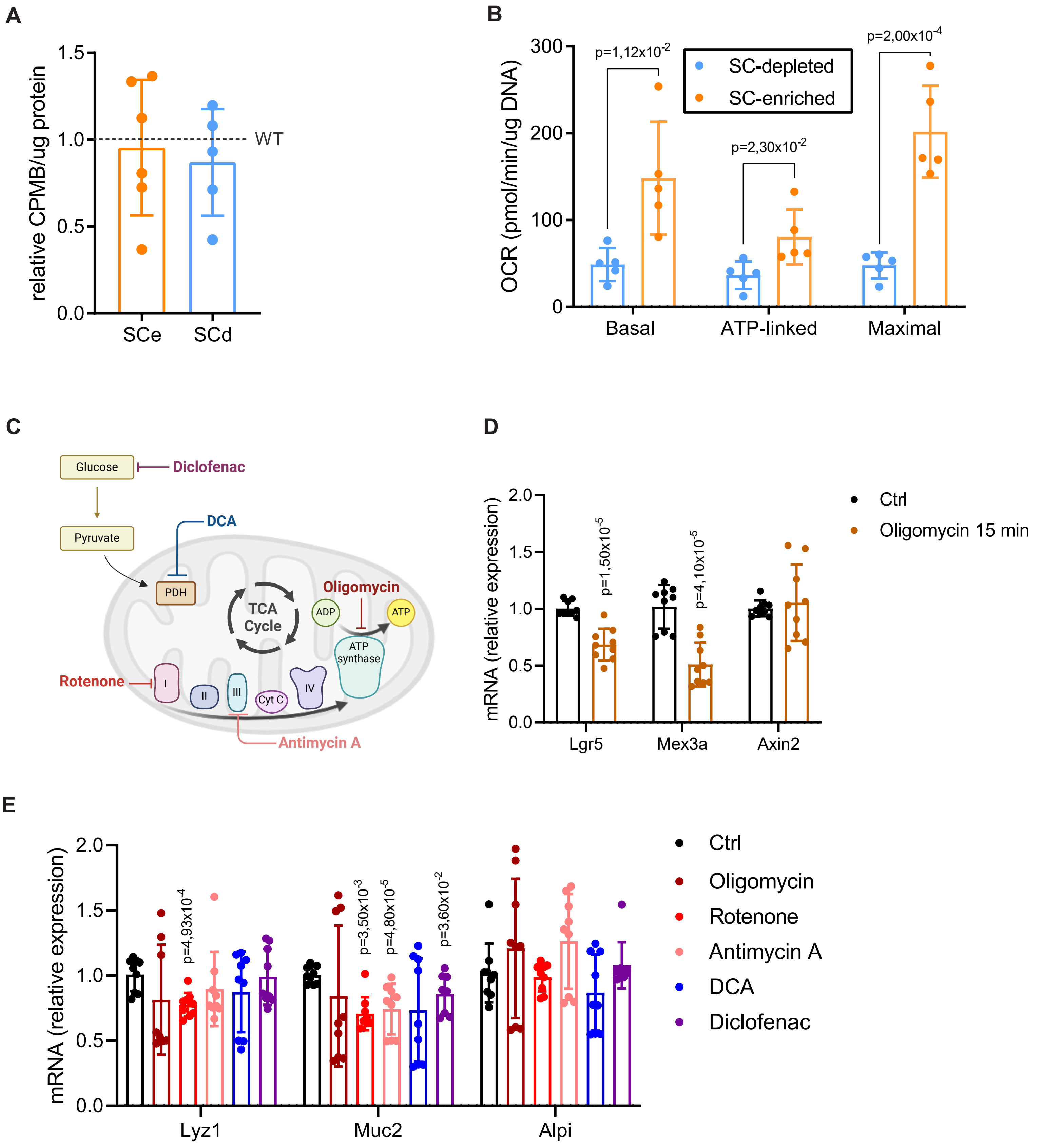

A
relative CPMB/ug protein
1.5
1.0
0.5
0.0
WT
SCe
SCd
B
OCR (pmol/min/ug DNA)
300
200
100
0
p=1,12x10-2
p=2,30x10-2
p=2,00x10-4
SC-depleted
SC-enriched
Basal
ATP-linked
Maximal
C
Glucose
Diclofenac
Pyruvate
DCA
PDH
TCA Cycle
Oligomycin
ADP
ATP
ATP synthase
Rotenone
I
II
III
Cyt C
IV
Antimycin A
D
mRNA (relative expression)
2.0
1.5
1.0
0.5
0.0
p=1,50x10-5
p=4,10x10-5
Ctrl
Oligomycin 15 min
Lgr5
Mex3a
Axin2
E
mRNA (relative expression)
2.0
1.5
1.0
0.5
0.0
p=4,93x10-4
p=3,50x10-3
p=4,80x10-5
p=3,60x10-2
Ctrl
Oligomycin
Rotenone
Antimycin A
DCA
Diclofenac
Lyz1
Muc2
Alpi

Fig. S4

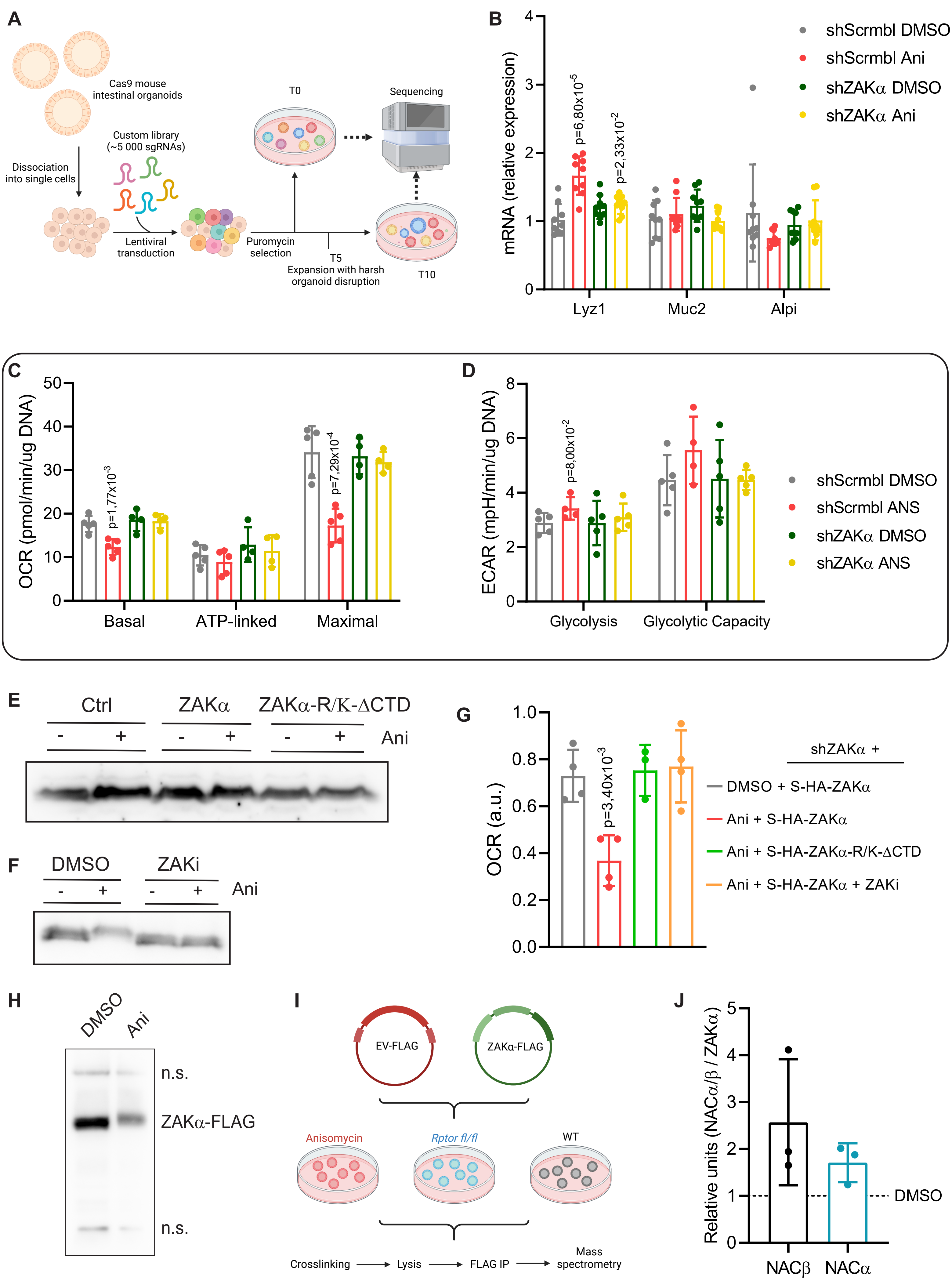

A
Cas9 mouse intestinal organoids
Dissociation into single cells
Custom library (~5 000 sgRNAs)
Lentiviral transduction
Puromycin selection
T0
T5
Expansion with harsh organoid disruption
T10
Sequencing
B
mRNA (relative expression)
p=6,80x10^-5
p=2,33x10^-2
Lyz1
Muc2
Alpi
shScrmbl DMSO
shScrmbl Ani
shZAKα DMSO
shZAKα Ani
C
OCR (pmol/min/ug DNA)
p=1,77x10^-3
p=7,29x10^-4
Basal
ATP-linked
Maximal
D
ECAR (mpH/min/ug DNA)
p=8,00x10^-2
Glycolysis
Glycolytic Capacity
shScrmbl DMSO
shScrmbl ANS
shZAKα DMSO
shZAKα ANS
E
Ctrl
ZAKα
ZAKα-R/K-ΔCTD
Ani
F
DMSO
ZAKi
Ani
G
OCR (a.u.)
p=3,40x10^-3
shZAKα +
DMSO + S-HA-ZAKα
Ani + S-HA-ZAKα
Ani + S-HA-ZAKα-R/K-ΔCTD
Ani + S-HA-ZAKα + ZAKi
H
DMSO
Ani
n.s.
ZAKα-FLAG
n.s.
I
EV-FLAG
ZAKα-FLAG
Anisomycin
Rptor fl/fl
WT
Crosslinking
Lysis
FLAG IP
Mass spectrometry
J
Relative units (NACα/β / ZAKα)
DMSO
NACβ
NACα

Fig. S5

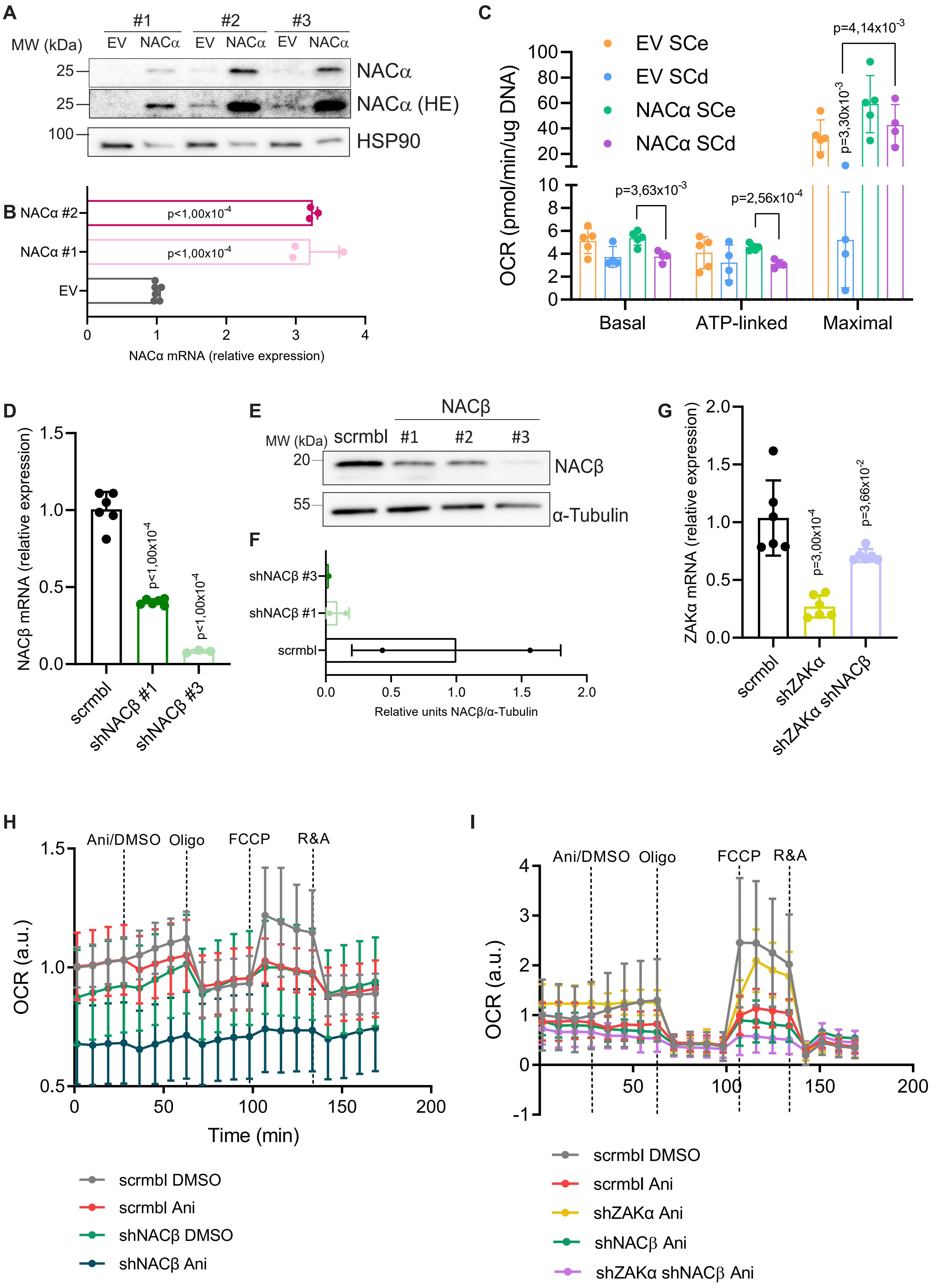

A
#1 #2 #3
MW (kDa) EV NACα EV NACα EV NACα
25 NACα
25 NACα (HE)
100 HSP90
B
NACα #2 p<1,00x10-4
NACα #1 p<1,00x10-4
EV
NACα mRNA (relative expression)
C
EV SCe
EV SCd
NACα SCe
NACα SCd
OCR (pmol/min/ug DNA)
p=3,63x10-3
p=2,56x10-4
p=3,30x10-3
p=4,14x10-3
Basal
ATP-linked
Maximal
D
NACβ mRNA (relative expression)
p<1,00x10-4
p<1,00x10-4
scrmbl
shNACβ #1
shNACβ #3
E
NACβ
MW (kDa) scrmbl #1 #2 #3
20 NACβ
55 α-Tubulin
F
shNACβ #3
shNACβ #1
scrmbl
Relative units NACβ/α-Tubulin
G
ZAKα mRNA (relative expression)
p=3,00x10-4
p=3,66x10-2
scrmbl
shZAKα
shZAKα shNACβ
H
Ani/DMSO Oligo FCCP R&A
OCR (a.u.)
Time (min)
scrmbl DMSO
scrmbl Ani
shNACβ DMSO
shNACβ Ani
I
Ani/DMSO Oligo FCCP R&A
OCR (a.u.)
scrmbl DMSO
scrmbl Ani
shZAKα Ani
shNACβ Ani
shZAKα shNACβ Ani

Fig. S6

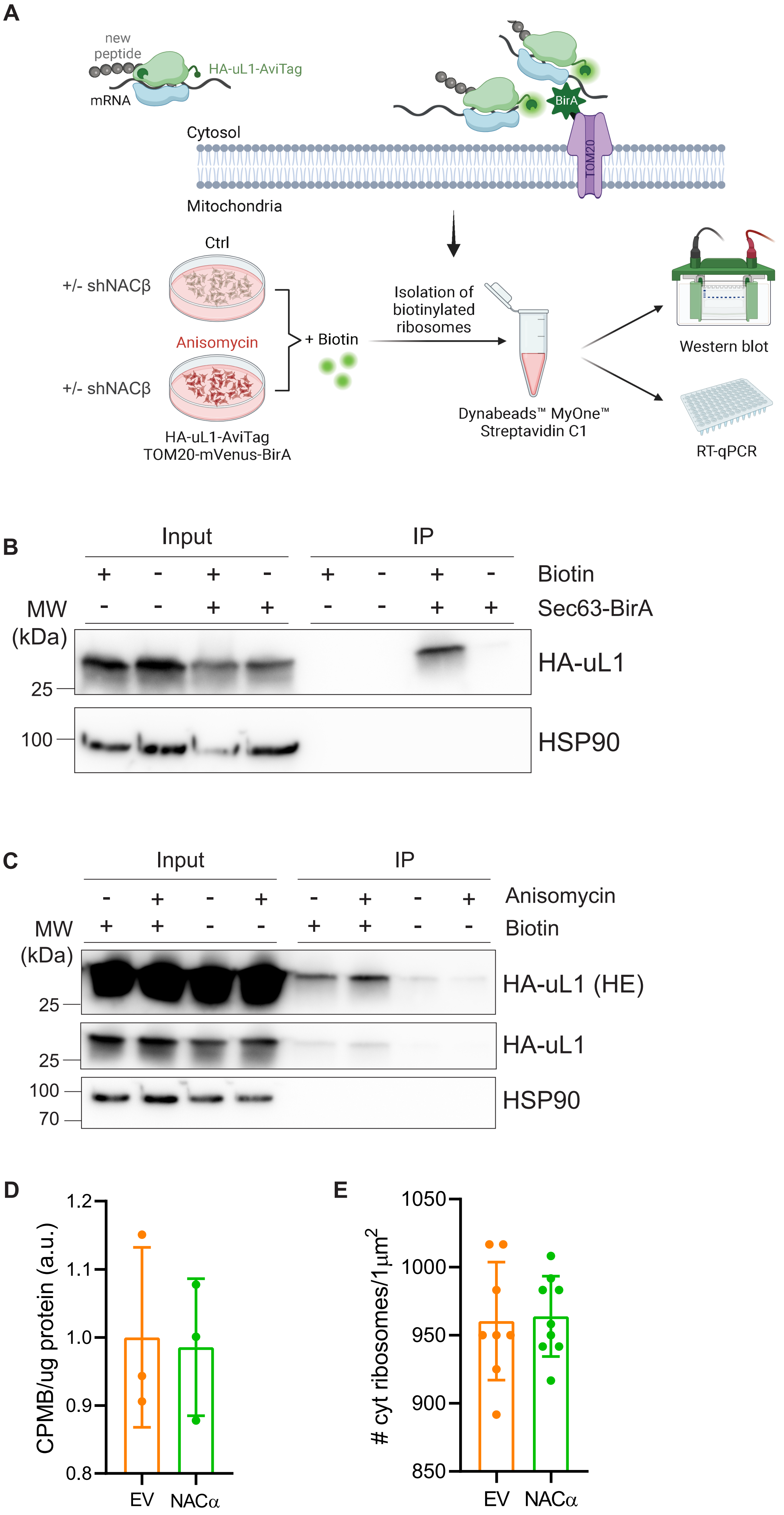
A
new peptide
mRNA
HA-uL1-AviTag
BirA
TOM20
Cytosol
Mitochondria
Ctrl
+/- shNACβ
Anisomycin
+/- shNACβ
HA-uL1-AviTag
TOM20-mVenus-BirA
+ Biotin
Isolation of biotinylated ribosomes
Dynabeads™ MyOne™ Streptavidin C1
Western blot
RT-qPCR
B
Input
IP
Biotin
Sec63-BirA
MW (kDa)
25
100
HA-uL1
HSP90
C
Input
IP
Anisomycin
Biotin
MW (kDa)
25
25
100
70
HA-uL1 (HE)
HA-uL1
HSP90
D
CPMB/ug protein (a.u.)
1.2
1.1
1.0
0.9
0.8
EV
NACα
E
cyt ribosomes/1μm²
1050
1000
950
900
850
EV
NACα

Fig. S1 - Quality Control plots for RiboSeq experiments performed in SCe (stem cell-enriched) and SCd (stem cell-depleted) organoids - Related to Figure 1

A) PCA plot for RiboSeq performed in SCe and SCd organoids. Three biological replicates were used for each condition.

B) Quality control (QC) plots for RiboSeq experiments performed in SCe and SCd organoids, generated by the RiboCode tool. QC plots include read length histograms and periodicity plots of mRNA-mapped reads separately for each sample.

C) Pie chart depicting the RiboCode ORF prediction statistics using the RiboSeq data from all samples. Raw numbers and percentages of different classes of ORFs are presented.

Fig. S2 - RNASeq analysis of SCe (stem cell-enriched) and SCd (stem cell-depleted) and organoids - Related to Figure 1

A) PCA plot for RNASeq performed in SCe and SCd organoids. Three biological replicates were used for each condition.

B) Gene Set Enrichment Analysis (GSEA) results based on RNASeq differential expression data comparing SCe to SCd organoids (n = 6 from 3 biological replicates). No significant differences are shown for signatures related to mitochondrial processes and glycolysis. p-values were determined using the *clusterProfiler* package.

C) Volcano plot showing the differential expression results of SCe and SCd comparison, highlighting that most genes involved in glycolysis and OXPHOS do not seem to change significantly between the two conditions. p-values were determined using the DESEQ2 package.

Fig. S3 - Total protein synthesis and seahorse quantification of SCe (stem cell-enriched) and SCd (stem cell-depleted) organoids and impact of metabolic inhibitors on differentiation markers expression - Related to Figure 1

A) Incorporation of ^{35}S-methionine shows no differences in total protein synthesis between SCe and SCd cultures. Mean and SD are shown (n = 6 (two biological replicates, each assessed in technical duplicates)). p-values were determined using a two-tailed t-test.

B) OCR analysis shows increased respiration in SCe compared to SCd organoids. Mean and SD are shown (n = 5 biological replicates). p-values were determined using a two-tailed t-test. Related to figure 1G.

C) Schematic diagram highlighting the metabolic targets of the different OXPHOS and glycolysis inhibitors used in Figure 1H. Created with BioRender.com.

D) RT-qPCR analysis of stem markers in WT organoids treated with oligomycin (1μM) for 15 minutes. *Hprt* was used as a housekeeping control. Mean and SD are shown (n = 9 (three biological replicates, each assessed in technical triplicates)). p-values were determined using a two-tailed t-test.

E) RT-qPCR analysis of genes related to differentiation of WT organoids treated with different OXPHOS (oligomycin, rotenone and antimycin A) and glycolysis (DCA and diclofenac) inhibitors for 24 hours. Hprt was used as a housekeeping control. Mean and SD are shown (n = 9 (three biological replicates, each assessed in technical triplicates)). p-values were determined using a two-tailed t-test.

Fig. S4 - Changes in cell fate and metabolism triggered by anisomycin treatment depend on ZAKα's ribosome-binding and kinase activity - Related to Figure 2

A) Scheme describing the CRISPR dropout screen workflow performed in mouse intestinal organoids and used to assess the essentiality of translationally-regulated hits obtained from previous RiboSeq experiment, for intestinal stem cells. Created with BioRender.com.

B) RT-qPCR analysis of genes related to differentiation of WT organoids treated with 1μM anisomycin for 30 minutes. *Hprt* was used as a housekeeping control. Mean and SD are shown (n = 9 (three biological replicates, each assessed in technical triplicates)). p-values were determined using a two-tailed t-test.

C) OCR analysis shows decreased respiration rates in WT organoids treated with 1μM anisomycin for 30 minutes, which is dependent on ZAKα. Mean and SD are shown (n ≥ 4 biological replicates). Related to Figure 2F.

D) Extracellular acidification rate (ECAR) analysis shows increased glycolytic rates upon treatment of WT organoids with 1μM anisomycin for 30 minutes, which is dependent on ZAKα. Mean and SD are shown (n ≥ 4 biological replicates). Related to Figure 2G.

E) Immunoblot of Phos-tag gel analysis shows that ZAKα phosphorylation upon anisomycin treatment is dependent on its ribosome-binding activity (R/K-ΔCTD).

F) OCR analysis shows rescue of the decrease in maximum respiration caused by anisomycin by a ribosome binding mutant version of ZAKα (S-HA-ZAKα-R/K-ΔCTD) and a specific ZAKα inhibitor, but not by wild-type ZAKα (S-HA-ZAKα). Mean and SD are shown (n ≥ 3 biological replicates).

G) Immunoblot of Phos-tag gel analysis shows that ZAKα phosphorylation upon anisomycin treatment is dependent on its kinase activity.

H) Immunoblot of Phos-tag gel analysis using a FLAG antibody confirms the phosphorylation of the FLAG-tagged ZAKα upon anisomycin treatment.

I) Schematic figure illustrating the approach used to explore potential new interactors of ZAKα in WT mouse intestinal organoids in two different conditions promoting ribosome impairment (*Rptor fl/fl* organoids and WT organoids treated with anisomycin), using rapid immunoprecipitation mass spectrometry of endogenous proteins (RIME). An empty vector expressing FLAG tag bait (EV-FLAG) was used as a control. Created with BioRender.com.

J) Western blot quantification (including the one shown in Figure 2K) shows an interaction of ZAKα with both NACα and NACβ in HCT116 cells, revealing this to be increased upon treatment with 1μM anisomycin for 30 minutes. EV-FLAG-infected cells were used as a control. The experiment was carried out in three biological replicates.

Fig. S5 - NAC overexpression and knockdown effects on respiration rates - Related to Figure 3

A) Western blot analysis of the levels of NACα in WT organoids cells overexpressing a EV-FLAG or a NACα-FLAG vector. HSP90 was used as a loading control. Experiments were done in three biological replicates.

B) RT-qPCR analysis of *NACα* expression in mouse intestinal organoids derived from two mice (#1 and #2) upon overexpression or transduction with an empty vector (EV). *Hprt* was used as a housekeeping reference. Mean and SD are shown (n = 3 (one biological replicate assessed in technical triplicates)). p-values were determined using a two-tailed t-test.

C) OCR analysis shows a rescue of respiration rates in SCd organoids overexpressing NACα. Mean and SD are shown (n ≥ 4 biological replicates). p-values were determined using a two-tailed t-test. Related to Figure 3A.

D) RT-qPCR analysis of *NACβ* expression in HCT116 cells upon knock down. *β-actin* was used as a housekeeping reference. Mean and SD are shown (n = 6 (two biological replicates assessed in technical triplicates) for shNACβ #1 and n = 3 (one biological replicate assessed in technical triplicates) for shNACβ #3). p-values were determined using a two-tailed t-test.

E) Western blot analysis of the levels of NACβ in HCT116 cells upon knock down. α-Tubulin was used as a loading control. Experiments were done in two biological replicates for shNACβ #1 and one for shNACβ #3.

F) Quantification of western blots shows a decrease in NACβ levels upon knock down. Mean and SD are shown (n = 2 biological replicates for shNACβ #1 all assessed in technical triplicates and n = 1 biological replicate for shNACβ #3). p-values were determined using a two-tailed t-test.

G) RT-qPCR analysis of *ZAK*α expression HCT116 cells upon knock down. *β-actin* was used as a housekeeping reference. Mean and SD are shown (n = 6 (two biological replicates assessed in technical triplicates)). p-values were determined using a two-tailed t-test.

H) OCR analysis shows decreased respiration rates in WT HCT116 cells treated with anisomycin (1μM, 30 minutes), upon NACβ knockdown and when combining both anisomycin treatment with NACβ knockdown. Mean and SD are shown (n = 5 biological replicates for each of the 2 independent shRNAs). Related to Figure 3G.

I) OCR analysis shows decreased respiration rates in WT HCT116 cells treated with anisomycin (1μM, 30 minutes) and the rescue of this decrease upon ZAKα knockdown. When NACβ is knocked down, the rescue of the anisomycin effect observed with the loss of ZAKα is not possible. Mean and SD are shown (n = 4 biological replicates). Related to Figure 3H.

Fig. S6 - NAC's influence on ribosome localization to the ER and overall protein synthesis

A) Schematic representation of the ribosome proximity labelling assay used for quantification of ribosomes in proximity of the outer mitochondrial membrane (OMM). HEK293T cells express the OMM protein TOM20 fused to the biotin ligase BirA, which recognizes a specific avidin acceptor fused to the HA-tagged ribosomal protein uL1 (HA-uL1) and biotinylates it upon biotin treatment. Biotinylated ribosomes were isolated using streptavidin beads and further western blot or RT-qPCR analysis was performed. Created with BioRender.com.

B) Western blot analysis confirms the efficiency of pulling down biotinylated ribosomes, measured by HA-uL1 levels upon treating cells with biotin. Experiments were carried out in one biological replicate.

C) Western blot analysis shows an increase in the number of biotinylated ribosomes, accessed by HA-uL1 levels, following anisomycin treatment. HSP90 serves as a loading control. Experiments were carried out in one biological replicate.

D) Incorporation of ^{35}S-methionine shows no differences in total protein synthesis between organoids overexpressing EV-FLAG and NACα-FLAG. Mean and SD are shown (n = 9 (three biological replicates). p-values were determined using a two-tailed t-test.

E) Quantification of cytosolic ribosomes in WT organoids overexpressing EV-FLAG and NACα-FLAG. Mean and SD are shown, corresponding to three regions (200 nm x 200 nm) from ≥ 8 different images. p-values were determined using a two-tailed t-test.

Table S1 – RNAseq and RiboSeq based differential analysis of gene expression and translational regulation metrics between SCe (stem cell-enriched) and SCd (stem cell-depleted) intestinal organoids.

Table S2 – sgRNA sequences for the CRISPR library that is designed for translationally regulated genes in SCe (stem cell-enriched) and SCd (stem cell-depleted) intestinal organoid comparisons.

Table S3 – Custom CRISPR library drop-out screening results, parsed from MAGECK-VISPR output (mle.rra.gene_summary).

Table S4 – Antibodies

Table S5 – Primer sequences

Table S6 – Oligo sequences used in ribosome profilling
